# Computationally Designed Epitope-Mediated Imprinted Polymers versus Conventional Epitope Imprints for the Detection of Human Adenovirus in Water and Human Serum Samples

**DOI:** 10.1021/acssensors.3c02374

**Published:** 2024-03-15

**Authors:** Ekin Sehit, Guiyang Yao, Giovanni Battocchio, Rahil Radfar, Jakob Trimpert, Maria A. Mroginski, Roderich Süssmuth, Zeynep Altintas

**Affiliations:** † Institute of Chemistry, Technical University of Berlin, Straße des 17. Juni 124, 10623 Berlin, Germany; ‡ Institute of Materials Science, Faculty of Engineering, Kiel University, 24143 Kiel, Germany; § Institute of Virology, Free University of Berlin, 14163 Berlin, Germany; ∥ Kiel Nano, Surface and Interface Science (KiNSIS), Kiel University, 24118 Kiel, Germany

**Keywords:** virus detection, epitope imprinting, QCM sensor, molecular dynamics, in silico-designed epitope-mediated adenovirus receptors

## Abstract

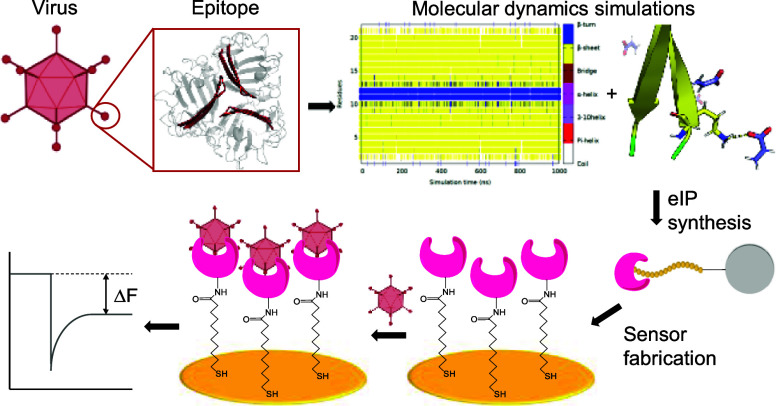

Detection of pathogenic viruses for point-of-care applications has attracted great attention since the COVID-19 pandemic. Current virus diagnostic tools are laborious and expensive, while requiring medically trained staff. Although user-friendly and cost-effective biosensors are utilized for virus detection, many of them rely on recognition elements that suffer major drawbacks. Herein, computationally designed epitope-imprinted polymers (eIPs) are conjugated with a portable piezoelectric sensing platform to establish a sensitive and robust biosensor for the human pathogenic adenovirus (HAdV). The template epitope is selected from the knob part of the HAdV capsid, ensuring surface accessibility. Computational simulations are performed to evaluate the conformational stability of the selected epitope. Further, molecular dynamics simulations are executed to investigate the interactions between the epitope and the different functional monomers for the smart design of eIPs. The HAdV epitope is imprinted via the solid-phase synthesis method to produce eIPs using in silico-selected ingredients. The synthetic receptors show a remarkable detection sensitivity (LOD: 10^2^ pfu mL^–1^) and affinity (dissociation constant (*K*_d_): 6.48 × 10^–12^ M) for HAdV. Moreover, the computational eIPs lead to around twofold improved binding behavior than the eIPs synthesized with a well-established conventional recipe. The proposed computational strategy holds enormous potential for the intelligent design of ultrasensitive imprinted polymer binders.

Several viral outbreaks have occurred since the start of the new millennium due to the uncontrolled spread of human pathogenic viruses (e.g., severe acute respiratory syndrome coronavirus (SARS-CoV) in 2002, influenza A virus H1N1 in 2009, Middle East respiratory syndrome coronavirus (MERS-CoV) in 2012, and Ebola virus in 2013).^1^ The most recent example is the pandemic of COVID-19 caused by SARS-CoV-2, leading to more than 7 million deaths and an enormous economic impact with an estimated value of US $1 trillion worldwide.^2,3^ Similarly, human adenovirus is another pathogenic virus which causes various diseases such as respiratory infections, conjunctivitis, bronchiolitis, gastroenteritis, and pneumonia.^4^ The adenoviruses are spread in the feces of infected patients and transmitted via the fecal–oral route through the contaminated food and water sources. The detection of adenovirus in contaminated water or human health samples demands highly sensitive recognition tools since the virus can lead to contamination or infection at very low doses.^5^

The management of viral outbreaks and controlling the spread of viral diseases require the successful detection of pathogenic viruses with rapid, accurate, and economical point-of-care (PoC) diagnostic devices. Furthermore, many preventive care methods such as sanitation, food inspection, and therapeutics critically need portable and affordable diagnostic tools for viruses. Currently, the viral diagnostics are firmly dependent on polymerase chain reaction (PCR) and enzyme-linked immunosorbent assay (ELISA) which are time-consuming, expensive, and involve laborious implementation, requiring medically trained staff.^6^ As a more user-friendly and cost-effective alternative, biosensors can be utilized in virus diagnostics. A number of studies have reported biosensing platforms targeting pathogenic viruses using antibodies,^7^ nucleic acids,^8^ and peptides^9^ as recognition elements. However, such receptors suffer from major drawbacks such as immense cost, long preparation times, low durability, denaturation under harsh environmental conditions, and enzymatic digestion.^10^

Molecularly imprinted polymers (MIPs) are enduring and inexpensive recognition materials that are utilized in biosensing applications. These fully synthetic receptors can bind to a specific target with high affinity due to the complementary cavities in the polymer matrix matching the analyte in terms of shape, size, and chemical functionality.^11^ A wide range of analytes have been targeted with MIP-based receptors including small molecules like glucose,^12^ pharmaceuticals,^13^ and endotoxins^14^ to large entities such as proteins,^15^ viruses,^16^ and bacteria.^17^ However, imprinting large biosystems is problematic due to the complex and unstable conformational structure leading to the formation of less efficient binding sites.^18^ The cost of a protein or a virus is exorbitant, especially when it is required in high amounts during the MIP synthesis. Such drawbacks are addressed by imprinting a small portion of the whole system (i.e., epitope) providing highly selective imprinted cavities which are capable of recognizing the entire entity.^19,20^

MIPs are also utilized in therapeutic applications, in which pathogenic viruses are targeted. Parisi and colleagues imprinted the receptor-binding domain protein (RBD) of SARS-CoV-2 via inverted microemulsion polymerization to inhibit its binding to the host cell receptor.^21^ In another work, antiviral nanoMIPs were fabricated targeting the viral glycan shield of SARS-CoV-2 preventing virus–receptor interaction while facilitating the clearance of virus via phagocytosis.^22^

Selection of an epitope is the key to generating high-affinity binding sites via epitope imprinting. In the previous works of our group, we have demonstrated that the polymer film imprinted with particularly stable epitopes could capture the targeted protein with four times higher sensitivity than the least stable epitope imprints.^20^ Another critical point is to employ functional monomers providing optimized interaction with the template to ensure a high affinity of the MIPs. Computational simulations are particularly useful tools to determine the optimum MIP composition since they shorten the trial-and-error-based experimental work in the lab.^14^

Herein, we combined computational approaches for both epitope selection and determination of polymer composition to obtain highly sensitive and specific epitope-imprinted polymers (eIPs) for human adenovirus detection. The proteins located in the outer shell (i.e., capsid) of human adenovirus 5 (HAdV) were investigated to determine the epitope, which was monitored afterward via molecular dynamics to evaluate its conformational stability. The epitope was further simulated with candidate functional monomers to select the monomers showing the highest affinity toward the epitope. The eIPs were synthesized using a computationally derived recipe and characterized by Fourier transform infrared spectroscopy (FT-IR) and dynamic light scattering (DLS) techniques. The in silico-designed eIPs were utilized on a compact and inexpensive quartz crystal microbalance device to fabricate eIP-QCM as a PoC device for viral diagnostics (Figure 1). The sensing platform was examined by electrochemical methods, atomic force microscopy (AFM), and fluorescence microscopy tools. The sensitivity, selectivity, and specificity of eIP-QCM were investigated. Moreover, the applicability for real sample analysis was demonstrated in HAdV-spiked tap water and human serum samples.

**Figure 1:**
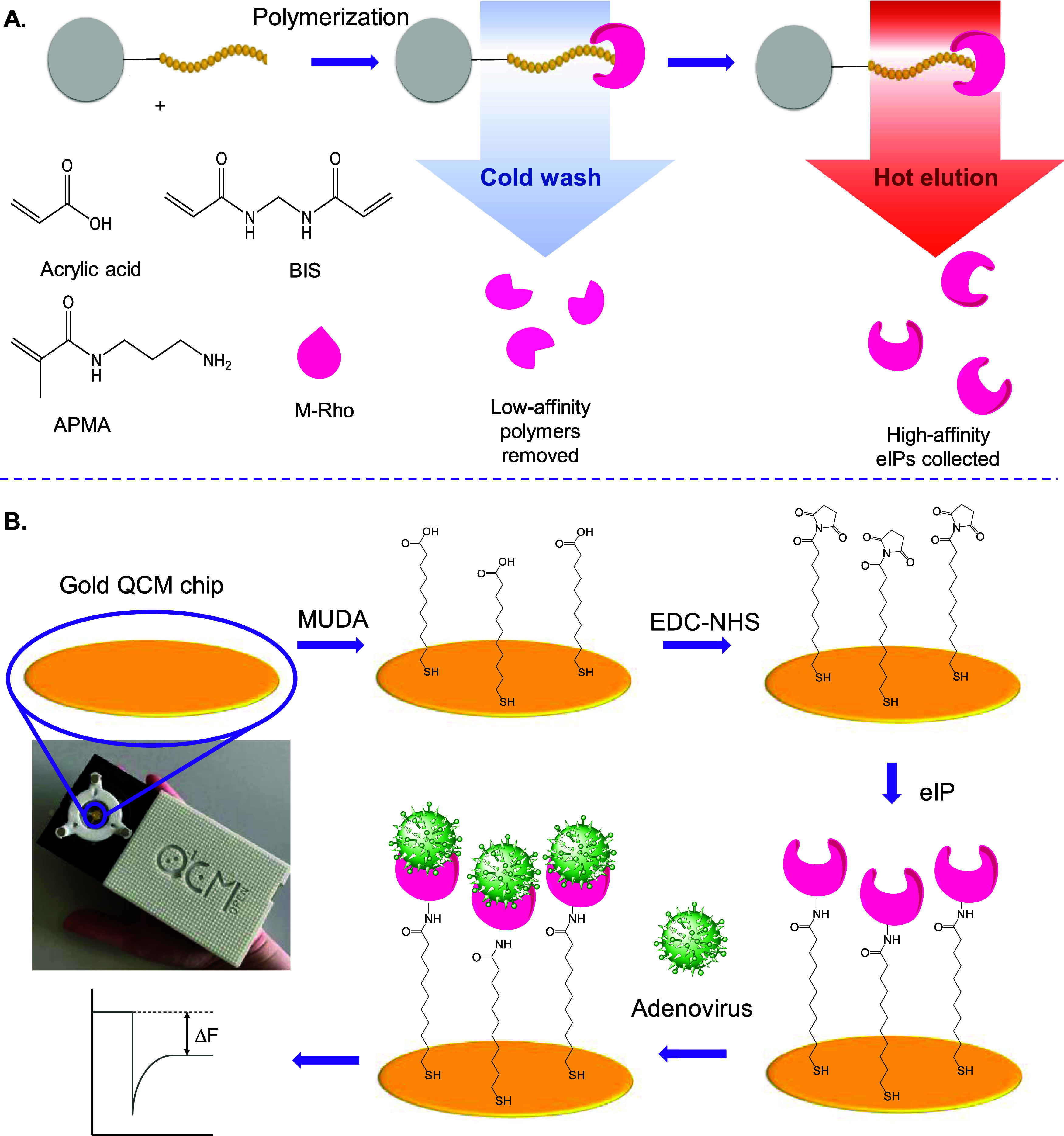
(A) Solid-phase synthesis of computationally designed eIPs. (B) Preparation of portable eIP-QCM biosensor and piezoelectric-based adenovirus detection via a knob–cavity interaction.

## Experimental Section

### Synthesis of eIPs Using Computational and Conventional Recipes

The eIPs were synthesized via the solid-phase synthesis method (Figure 1A) in which the template epitope was immobilized on a glass support prior to polymerization.^23^ For solid-phase synthesis, 60 g glass beads were boiled in 2 M NaOH for 15 min, and the surface was further silanized with 2% v/v (3-aminopropyl)triethoxysilane (APTES) solution in dry toluene with overnight incubation. The silanized beads were incubated with 7% v/v glutaraldehyde in 10 mM phosphate-buffered saline (PBS) for 2.5 h. In the meantime, 10 mg of the epitope was dissolved in 5 mL of methanol and further diluted to 40 mL with PBS. The epitope solution was incubated with glutaraldehyde-functionalized beads overnight at room temperature for template immobilization. The computationally derived polymerization recipe was prepared using 5.33 μL (77.7 × 10^–6^ mol) of acrylic acid, 0.24 mg (1.56 × 10^–6^ mol) *N*,*N*′-methylenebis(acrylamide) (BIS), 8.9 mg (46.98 × 10^–6^ mol) *N*-(3-aminopropyl)methacrylamide (APMA), and 0.9 mg (1.3× 10^–6^ mol) methacryloxyethyl thiocarbamoyl rhodamine B (M-Rho) in 24 mL of the polymerization mixture. Once the mixture is sonicated and purged with N_2_ for 30 min, it was combined with the epitope-immobilized glass beads. The polymerization was initiated with the addition of 3.56 mg of ammonium persulfate (APS) in aqueous solution and 1.31 μL of *N*,*N*,*N*′,*N*′-tetramethylethylenediamine (TEMED). After 2 h of polymerization, the beads were transferred into a solid-phase extraction column and washed with ultrapure water at 15 °C to remove weakly bound low-affinity eIPs, oligomers, and residual monomers. The high-affinity eIPs were eluted with ultrapure water at 65 °C and freeze-dried before storage. Conventional eIPs were synthesized by imprinting the HAdV epitope using a previously reported recipe, which has been used by several prominent research groups.^24−27^ Control eIPs were synthesized for the 22-mer p53 epitope to perform selectivity studies. In addition, nonimprinted polymers (NIPs) were synthesized using same procedure as that for eIP synthesis via solid-phase synthesis without the addition of the template epitope to further confirm the selectivity of the eIPs.

Fourier transform infrared (FT-IR) spectroscopy and dynamic light scattering (DLS) measurements were carried out to study the properties of the eIP particles. FT-IR measurements were conducted using a Vertex 70 (Bruker, Germany) system with dry eIP samples in the attenuated total reflectance mode. For DLS characterization, 1 mg mL^–1^ eIPs aqueous solution was measured via backscatter mode (173°) in triplicate at 25°C using Zetasizer Ultra (Malvern Panalytical Ltd., U.K.). Also, zeta-potential evaluation of eIPs was carried out using the same device (*n* = 3). To determine the actual size of eIPs, high-resolution transmission electron microscopy (HRTEM) measurements were performed on a FEI Tecnai F30 G^2^ STWIN system (300 kV, FEG) after dilution in ultrapure water and by gently dripping them on a lacey carbon Cu TEM grid for sample preparation.

### Preparation of eIP-QCM Sensor

The sensor fabrication procedure is outlined in Figure 1B. The gold surface of QCM sensor chips (Novaetech S.r.l.) was modified with 2 mM 11-mercaptoundecanoic acid (MUDA) solution in ethanol via overnight incubation. The carboxylic acid groups were activated with 0.2 M 1-ethyl-3-(3-(dimethylamino)propyl)carbodiimide hydrochloride (EDC) and 0.05 M *N*-hydroxysuccinimide (NHS) mixture for 4 min, followed by the injection of 0.1 M sodium acetate buffer (pH 5.0, 10 mM). The eIPs were dissolved in 10 mM PBS buffer (pH 7.4) and sonicated at the desired concentration prior to incubation on the sensing platform for 20 min, during which the frequency readout of the QCM was stabilized. Following eIP immobilization, the sensor surface was treated with 100 μg mL^–1^ bovine serum albumin (BSA) for 2 min and 0.1 mM ethanolamine solution for 4 min as the blocking agents to prevent unspecific binding events. For selectivity studies, NIP-conjugated QCM platforms were prepared using the same procedure as that for eIP-QCM sensor preparation.

### Characterization of eIP-QCM Sensor

The new QCM sensor modified with 2 mM MUDA was examined before and after eIP immobilization on the surface by employing microscopic techniques. The atomic force microscopy (AFM) measurements were performed by a NanoWizard II system (JPK Instruments AG., Germany) to obtain 2D surface topography, the root-mean-square (RMS) roughness, and phase images. The measurements were acquired at room temperature with dry samples in intermittent contact mode employing TAP300 GD-G cantilevers from Budget Sensors (Innovative Solutions Bulgaria Ltd., Bulgaria). The resonance frequency of the cantilevers was 200–400 kHz with a force constant of 40 N/m. The scanning rate throughout the measurements was kept at 0.3–0.2 Hz. The samples were investigated in 10 × 10 μm scanning areas, and the JPKSM Data Processing software was used for data evaluation. Fluorescence microscopy images were recorded using a Keyence compact fluorescence microscope BZ-X810 (Keyence, Osaka, Japan) under 10× magnification and further analyzed with BZ-X800 Analyzer software.

### Virus Detection Assays

The portable eIP-QCM sensor was utilized in a semistatic way during the whole study. The samples were manually pulled inside the measurement chamber (ca. 100 μL) from the outlet tubing with a syringe, while the inlet tubing was inserted into the reservoir filled with the desired solution. Once the solution covered the chip surface without air bubbles, the injection was stopped. Third overtone of the resonance frequency was followed throughout the study as it provides a more stable frequency reading compared to the fundamental mode. Prior to the analyte injection, the eIP-QCM sensor surface was treated with PBS buffer (10 mM, pH: 7.4) for 10–15 min until a stable signal was recorded as a reference. The analyte solutions prepared in PBS were inserted into the chamber sequentially starting from the lowest concentration to the highest. After 7–10 min of the insertion of the analyte, a stable point was reached, and the surface was rapidly washed with PBS. During the syringe injections, a well-like signal formation was realized due to the pressure change, and the signal was restored when the injection was stopped. The frequency change response (Δ*F*) was calculated by taking the difference between the reference signal and the stabilized signal of the analyte. For a more reliable analysis, both target rebinding and reference signals were taken as average frequency readings in the stabilized region

### HAdV Detection in Tap Water and Human Serum

The eIP-QCM sensor performance was evaluated in spiked tap water and human serum samples to evaluate the sensor performance in real applications. HAdV-spiked tap water samples were prepared in the concentration range of 10^2^–10^7^ PFU mL^–1^, starting with a 100 μL of 10^8^ PFU mL^–1^ stock HAdV solution. The final 1 mL of HAdV-spiked samples included 90% tap water and 10% PBS. Prior to the detection of HAdV in tap water samples, a reference solution of an unspiked tap water–PBS (9:1 v/v) was injected and monitored for 20 min to be taken as a reference signal.

For biosensing experiments in serum, serially diluted rebinding solutions were prepared by diluting 100 μL of 10^8^ PFU mL^–1^ stock HAdV solution in 890 μL of PBS and 10 μL of human serum to prepare 10^7^ PFU mL^–1^ in diluted human serum. This solution was then further serially diluted into 900 μL of 1% human serum in portions of 100 μL to obtain rebinding samples in the concentration range of 10^2^–10^7^ PFU mL^–1^ in 100 times diluted serum media. A solution of 1% human serum without HAdV was used as the reference solution before the insertion of spiked samples into the eIP-QCM sensor. After a rapid wash with PBS, starting from the lowest concentration, spiked samples were inserted onto the measurement chamber and monitored for 10 min at room temperature.

## Results and Discussion

### Adenovirus Epitope Selection

**Figure 2:**
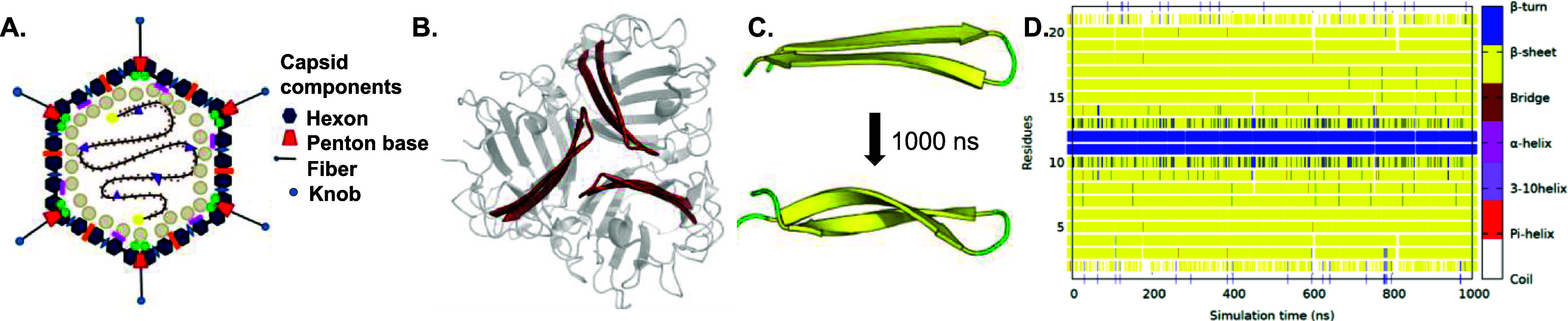
(A) Structure of adenovirus with capsid components. Reproduced under the terms of the CC-BY 4.0 license.^4^ (B) Adenovirus fiber knob structure (PDB: 6HCN), with 22-mer epitope highlighted. (C) Slight twisting behavior of the epitope during MD simulations. (D) Secondary structure evolution plot of the epitope during 1000 ns MD simulations.

Epitope–receptor binding event requires accessibility between the two moieties to ensure an efficient recognition. In order to design a receptor with high affinity, the HAdV structure was initially studied (Figure 2). HAdV carries a linear double-stranded DNA as the genetic material stored in the inner core complexed with histon-like proteins, while the outer part of the virus is covered with a protein shell called capsid.^28^ HAdV accommodates several proteins named as hexon, penton base, and fiber within its icosahedral-shaped capsid (Figure 2A).^29^ Among these proteins, the fiber knob protein is the most favorable capsid component since it extends out of the virus, providing a highly accessible surface for potential receptor–virus binding events. In addition, HAdV binds to its host cell receptor using the fiber knob protein.^29^ Therefore, fiber knob protein (PDB: 6HCN) was the focus of computational investigation. Since β-sheet conformation is highly conserved with low amino acid variability, three candidate epitopes with β-sheet structure were initially considered while omitting the areas with no assigned secondary structures.^30^ These epitopes were further evaluated by considering the electron density fit, geometrical quality, and surface accessible area (Figure S1). Epitope 2 and Epitope 3 depicted several outliers (i.e., ASN482, ASN562, ILE564, and GLU566) indicating the proposed structures not matching with the empirical data. Furthermore, Epitope 3 contained several areas without a distinct secondary structure. On the other hand, Epitope 1 presented significant geometrical quality with a good surface accessibility. As a result of this preliminary evaluation, the 22-mer Epitope 1 with the amino acid sequence of AKLTLVLTKCGSQILATVSVLA was selected (Figure 2B). The conformational stability of the selected peptide was further studied with molecular dynamics. The simulations revealed that the initial β-sheet epitope is preserved with a slightly twisted conformation (Figure 2C). The secondary structure evaluation demonstrated that the overall hairpin conformation remained secure throughout the 1000 ns long simulations with a low root-mean-square deviation (RMSD) of 1.84 ± 0.48 for the last 500 ns of simulation (Figure 2D). Such a negligible deviation was expected as the peptide is now removed from the whole protein structure.

### Computational Design of eIPs

Computational tools were employed to monitor the interactions between the epitope and monomer candidates to determine the optimum polymer composition without requiring laborious, expensive, and time-consuming experimental studies. Herein, the novel eIPs were designed in silico to acquire high-quality and tailor-made synthetic protein binders specific for the targeted virus. In order to achieve this, five candidate functional monomers (i.e., acrylic acid, methacrylic acid, 4(5)-vinyl imidazole, acrylamide, and methacrylamide) that are commonly used in the imprinting field were simulated with the HAdV epitope. Initially, each functional monomer was simulated separately, and the number of hydrogen bonds formed with the epitope was screened. The resulting plots revealed that 4(5)-vinyl imidazole (Figure S4), acrylamide (Figure S5), and methacrylamide (Figure S6) formed very few hydrogen bonds with the epitope. On the other hand, acrylic acid (Figure S2) and methacrylic acid (Figure S3) established a higher number of interactions with the epitope, suggesting that they are suitable options for further computational investigations. In the next step, different ratios of acrylic acid and methacrylic acid were simulated to determine the optimum synthesis conditions. The computational evidence exhibited that acrylic acid formed a higher number of bonds with the epitope (112 salt bridges and 2 double salt bridges) than methacrylic acid (88 salt bridges and 5 double salt bridges). Furthermore, both acrylic acid and methacrylic acid formed more bonds with the epitope when they were the only functional monomer in comparison to the case where two functional monomers were used in combination. This is attributed to the competition of similar functional groups of two monomers for the same binding sites of the epitope. Moreover, among these two potential functional monomers for experimental studies, the number of contacts was higher for acrylic acid (Figure S7). As a result of the computational evaluations, acrylic acid was selected as the functional monomer of the computational recipe.

### eIP Synthesis and Characterization

Prior to eIP synthesis, the adenovirus epitope with 22 residues was synthesized and characterized (Figure S8A,B). The selected peptide sequence accommodated many hydrophobic residues (e.g., leucine, valine, and alanine) resulting in a hydrophobic peptide chain which hinders the effective synthesis of the eIPs. In order to tackle this issue, the hydrophilicity of the epitope was improved by adding two aspartic acid residues as a side chain to the lysine residue which is adjacent to alanine in the N-terminal (Figure S8A). The epitope-imprinted nanoparticles were synthesized using a solid-phase synthesis method (Figure 1A). The low hydrophilicity of the epitope was addressed by dissolving the peptide initially in an organic solvent (i.e., methanol), which was then diluted with PBS buffer during the synthesis procedure. The epitope:functional monomer molar ratio, which was determined in the computational simulations, was maintained at 1:20 for epitope and acrylic acid.

In addition to the computationally determined acrylic acid functional monomer, N,*N*′-methylenebis(acrylamide) (BIS) bifunctional monomer was used as the cross-linking agent, while *N*-(3-aminopropyl)methacrylamide (APMA) was included into the polymerization mixture to introduce primary amine functionalities to the eIP particles. The incorporated primary amine groups were later utilized for eIP immobilization on gold QCM surface via carbodiimide coupling.^31^ The fluorescent dye-carrying monomer methacryloxyethyl thiocarbamoyl rhodamine B (M-Rho) was used to obtain fluorescence properties to aid fluorescence microscopy investigations. The synthesized nanoparticles were characterized by Fourier transform infrared (FT-IR) spectroscopy to confirm the successful synthesis of the eIPs (Figure S9). The FT-IR spectrum showed a broad peak at 3550–3200 cm^–1^ which was assigned to OH from carboxylic acid and NH from the amide and amine groups.^32^ The peak at 1652 cm^–1^ was attributed to the carbonyl group of both carboxylic acid and amide groups in the polymer structure, while the peak at 2320–2370 cm^–1^ resulted from O=C=O stretching of CO_2_ in the atmosphere.^33^ The incorporation of rhodamine dye into the polymer was confirmed by the presence of the peak at 786 cm^–1^ that resulted from aromatic ring stretching.^34^ The overall spectrum shows the expected characteristic peaks of eIPs. The size distribution of the polymeric nanoparticles was investigated by employing DLS measurements, revealing that eIPs have a hydrodynamic diameter of 145.1 ± 2.26 nm (Figure S8C). In addition, the solution contains 0.16% large polymeric aggregates with an average size of 1765 ± 150 nm. However, the number intensity plot shows that the aggregates are present only in a negligible amount compared to the eIPs (Figure S8D). High-resolution transmission electron microscopy (HRTEM, FEI Tecnai F30 G^2^ STWIN, USA) imaging was performed for a detailed investigation of eIPs (Figure S10). The eIPs were observed as globular porous structures with an average diameter of 71.8 ± 8.12 nm. Furthermore, the zeta potential of eIPs was investigated to understand their stability in liquid medium. The average zeta potential for eIPs was determined as −20.46 ± 1.14 mV (Figure S8E).

### Preparation of eIP-QCM Sensor

The eIPs were conjugated on an 11-mercaptoundecanoic acid (MUDA)-coated gold chip to be utilized as the synthetic ligands in the portable virus sensing platform. Following the activation of the carboxyl groups of MUDA via 0.2 M 1-ethyl-3-(3 dimethylaminopropyl)carbodiimide hydrochloride (EDC) and 0.05 M *N*-hydroxysuccinimide (NHS) mixture, eIPs were immobilized onto the sensor through the primary amine functionalities introduced with the APMA monomer. Three different eIP concentrations (e.g., 0.5, 1, and 1.5 mg mL^–1^) were examined initially to optimize the sensor configuration (Table S1): 0.5 mg mL^–1^ showed a nonsignificant decrease of approximately 3.66 ± 2.68 Hz, meaning insufficient eIP immobilization; 1 mg mL^–1^ gave a frequency difference of 60.11 ± 4.78 Hz that resulted in the highest amount of eIP particle immobilization on the surface; although 1.5 mg mL^–1^ was more concentrated, it showed a frequency decrease of 18.78 ± 2.99 when applied onto the QCM surface. This is most probably caused by the aggregation of the eIP particles at a high concentration, leading to lower probability of interaction with the surface.

**Figure 3:**
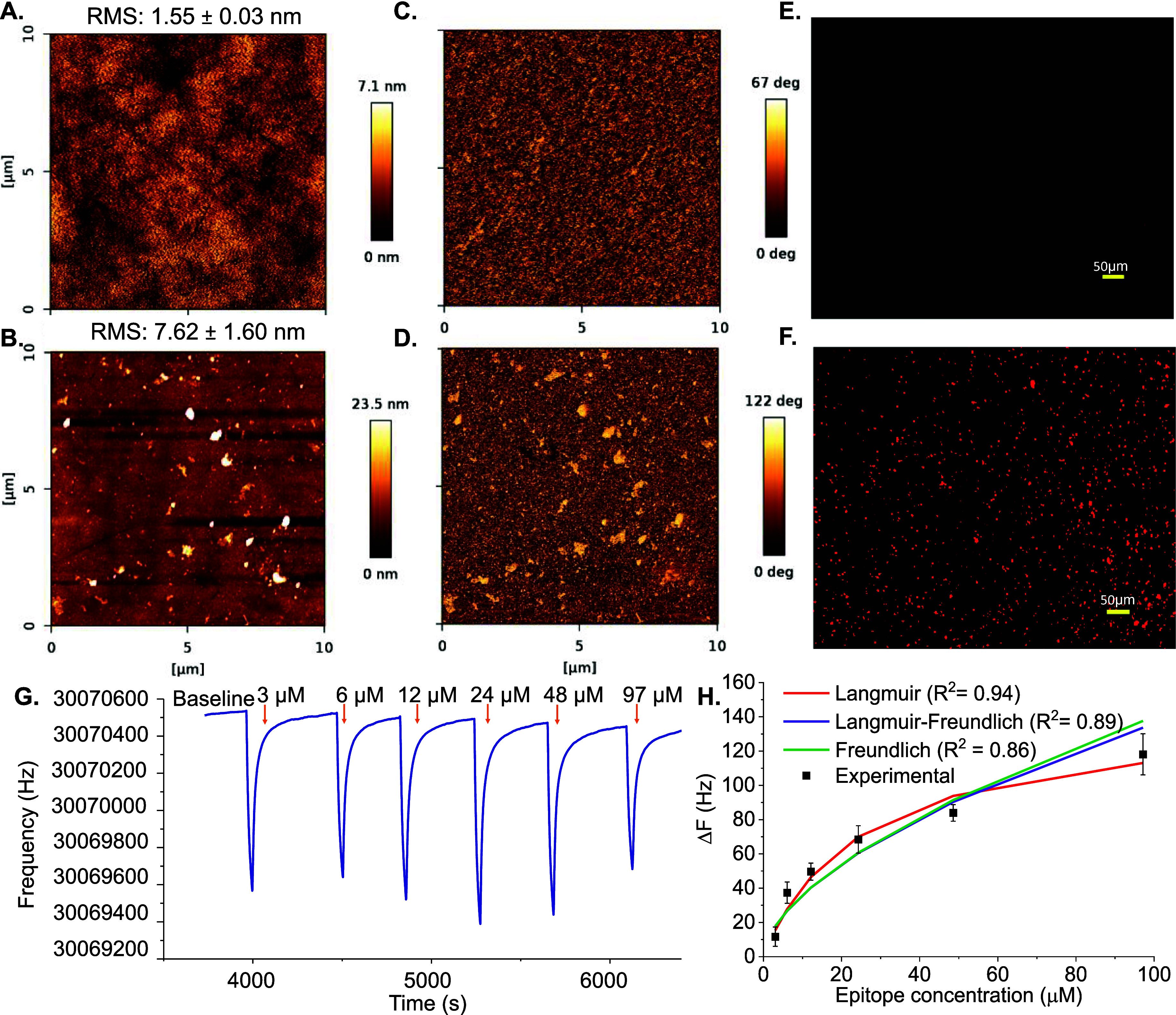
AFM (A) 2D height image, and (C) phase image of MUDA-coated QCM surface. (B) 2D height image, and (D) phase image of eIP-QCM sensor after eIP immobilization. Fluorescence microscopy images (E) before and (F) after eIP immobilization on MUDA-functionalized surface (10× magnification). Detection of templated epitope with eIP-QCM. (G) Real-time QCM sensogram shows the frequency pattern when epitope is introduced onto the surface (3–97 μM). (H) Concentration-dependent binding data were fitted into three different binding models (i.e., Langmuir, Freundlich, and Langmuir–Freundlich).

Since 1 mg mL^–1^ of eIPs exhibited the highest mass adsorption on the surface, it was used for sensor preparation throughout the study. The QCM chip surface was characterized before and after eIP immobilization by employing AFM and fluorescence microscopy to confirm the successful coverage of the surface face with the synthetic receptors. Two-dimensional surface height and phase images were obtained before and after eIP immobilization on the QCM surface in 10 × 10 μm^2^ scanning area (Figure 3). The 2D surface height image of the MUDA-coated gold surface was smooth and uniform with a low RMS roughness value of 1.55 ± 0.03 nm, which was expected for the self-assembled monolayer formed with MUDA (Figure 3A). Following the eIP conjugation step, an apparent alteration in the 2D surface image was observed as light-colored areas attributed to eIPs attached on the gold substrate in the aggregates (Figure 3B). In addition to the aggregates, the eIPs scattered onto the surface can also be observed. Due to the substantially large size, the RMS roughness value was increased almost fivefold to 7.62 ± 1.60 nm upon binding. Phase images were acquired for chemical mapping of the surface before and after eIP conjugation.^18^ The phase image of the MUDA-modified QCM crystal showed a homogeneous distribution of the chemical functionalities throughout the surface (Figure 3C). Following the eIP modification, the light-yellow phase was observed as scattered regions on the substrate which were assigned to eIPs (Figure 3D). Such a significant distinction before and after eIP conjugation agrees with the 2D height, confirming the successful immobilization of eIPs on the sensing surface. Fluorescence microscopy studies were also performed for the characterization of fluorescent dye-incorporated eIP particles on the QCM sensor. Figure 3E exhibits no significant fluorescent signal coming from the MUDA-coated sensor because the self-assembled monolayer does not have fluorescence characteristics. On the other hand, after immobilization of the eIPs on the QCM chip (Figure 3F), the presence of fluorescent eIPs as agglomerated particles on the surface was clearly observed. The presence of polymeric agglomeration on a sensor chip may result in elevated binding responses on a sensor surface. To avoid such a problem that may also lead to unspecific binding results, an appropriate blocking reagent should be used in sensor development. Herein, two distinct reagents, i.e., BSA and ethanolamine, were utilized for sensor surface preparation.

### Bioassays with eIP-QCM Sensor

After successfully confirming the sensor fabrication via microscopic techniques, the sensing performance of the eIP-QCM platform was evaluated. The successful formation of epitope-imprinted cavities was confirmed by detection assays targeting the template epitope. The eIP-QCM sensor was exposed to epitope solutions within the concentration range of 3–97 μM in 10 mM PBS buffer starting from the lowest concentration. While the peptide molecules bound to the cavities of immobilized eIP-QCM surface, the mass of the deposited film on the sensor was increased leading to a reduced resonance frequency of the piezoelectric crystal, as stated by Sauerbrey (Equation S1). The change in the resonance frequency (Δ*F*) of the sensor was used as the response to realize the concentration-dependent binding behavior. Δ*F* was calculated for each concentration as the difference between the average reference signal and the average stabilized signal observed after analyte injection. The frequency of eIP-QCM decreased stepwise, while the introduced analyte concentration increased (Figure 3G). The concentration-dependent frequency change was plotted to investigate the binding behavior between the analyte and the plastic receptors (Figure 3H). The binding isotherm data were fitted into three different binding models namely Langmuir, Freundlich, and Langmuir–Freundlich (LF) hybrid model.^35^ The highest *R*^2^ value, indicating the best fit between the experimental data and the model, was obtained with the Langmuir binding model. The Langmuir binding model assumes complete homogeneity of the binding sites; therefore, such a fitting shows that the binding sites formed within the polymeric matrix of eIPs are homogeneous similar to monoclonal antibodies. Upon the data fitting to the model, the dissociation constant (*K*_d_) was calculated assuming the Langmuir model as 2 × 10^–5^ M.^11,35^

**Figure 4:**
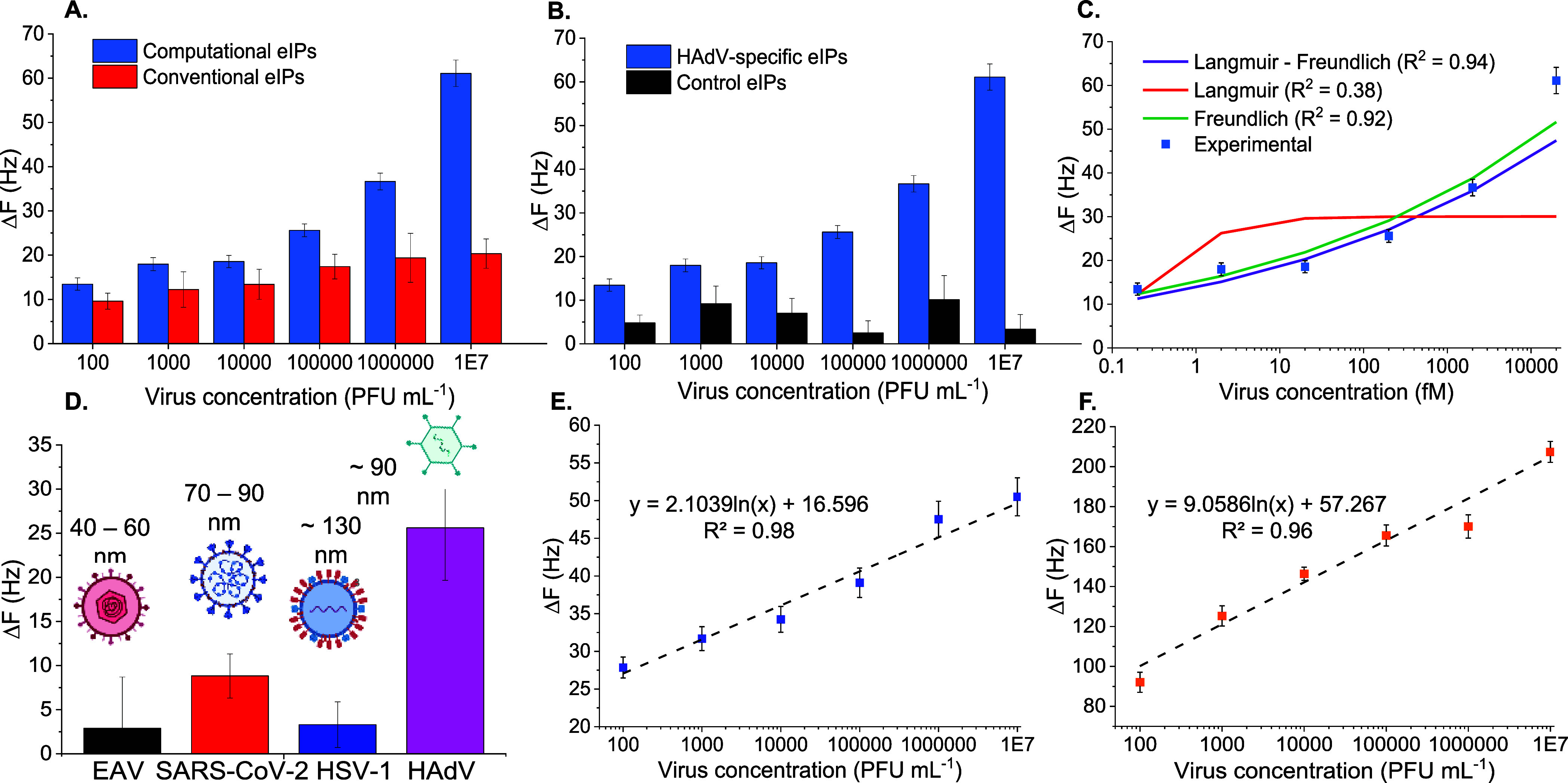
(A) HAdV detection performance of computationally designed eIPs in comparison to eIPs synthesized with conventional recipe. (B) HAdV detection with computationally derived HAdV-specific eIPs compared to control eIPs imprinted with another template (22-mer peptide of p53). (C) HAdV binding isotherm with computational eIPs fitted into three binding models (i.e., Langmuir, Freundlich, and Langmuir–Freundlich). (D) Cross-reactivity of different pathogenic viruses (EAV, SARS-CoV-2, and HSV-1) on HAdV-specific eIP-QCM sensor. HAdV detection in spiked (E) tap water and (F) human serum for a concentration range of 10^2^–10^7^ PFU mL^–1^.

Once the efficient binding between the imprinted cavities and the template is confirmed, the eIP-QCM sensor was evaluated for HAdV detection in buffer medium (Figure 4A). HAdV particles suspended in a 10 mM PBS buffer solution were introduced onto the eIP-QCM sensor in a concentration range of 10^2^–10^7^ PFU mL^–1^. The frequency change observed upon analyte injection was plotted against virus concentration in logarithmic scale. Δ*F* increased gradually with an increasing concentration since the HAdV particles bound from the fiber knobs to the eIPs, leading to increased mass on the microbalance. An experimentally determined limit of detection was revealed as 10^2^ PFU mL^–1^. Considering that there are an estimated 20–100 noninfectious viral adenovirus units for every plaque-forming viral particle, 10^2^ PFU mL^–1^ would correspond to a detection limit of 0.2 fM.^36^ The remarkable sensitivity of the computational eIPs was attributed to the tailor-made recipe designed for the specific epitope. Furthermore, the performance of the computationally derived recipe was compared to the eIPs synthesized with the conventional recipe which was previously utilized for imprinting peptides and viruses.^24,37^ Although the conventional eIPs showed a concentration-dependent increase in the sensor signal for HAdV, the obtained signal was lower than what is observed for computational eIPs at every concentration. The computational eIPs showed a 1.77 times higher signal than that of conventional eIPs on average. Such a stark difference in the sensor readout indicates that the computationally designed polymeric materials provided a superior sensitivity for HAdV while utilizing only one type of functional monomer instead of multiple varieties, proving that the in silico design leads to the production of more sensitive, efficient, and cost-effective MIPs.

Moreover, the dissociation constant *K*_d_ was calculated to investigate the affinity of eIPs toward HAdV. The concentration-dependent HAdV binding plot was evaluated using three different adsorption models, revealing that the LF binding model was the most suitable, with the *R*^2^ value of 0.94 (Figure 4C). The LF hybrid model combines both Langmuir and Freundlich adsorption models together in eq 1 in which *N*_t_, *a*, and *m* are the fitting parameters for bound (B) and free (F) analyte molecules. LF is commonly applied to molecularly imprinted polymer as it takes the heterogeneity of the binding sides into consideration by incorporating the parameter *m* into eq 1.^35^
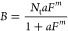
1

As the constant *m* deviates from 1 to 0, the heterogeneity of the binding system increases, and the model approaches from the Langmuir model to the Freundlich model.^35^ The fitting of the experimental plot in Figure 4C indicates that the LF model fitting approximates toward the Freundlich model with the heterogeneity constant *m* = 0.124. The binding sites exhibit a homogeneous distribution of binding energies for the epitope; however, when the epitope is a part of a large bioentity such as virus, the cavities of the eIPs show a heterogeneous binding affinity. The differentiation of the binding manner is caused by the change in the nature of the analyte.^39^ Since the imprinted epitope is a part of a hierarchical and complex structure within the virus capsid which accommodates various moieties (e.g., lipids and glycoproteins), the binding event is altered to a heterogeneous system.^16^ The average affinity constant *K*_0_ was calculated as 2.22 × 10^14^ M^–1^ based on the previously described heterogeneous binding systems.^38^ Employing the inversely proportional relation between *K*_0_ and *K*_d_, *K*_d_ was calculated as 4.48 × 10^–15^ M.^11^ Furthermore, the affinity of eIPs was also evaluated using a surface plasmon resonance (SPR) sensor (Biacore X100, Cytiva, Germany) which allows the observation of binding kinetics. The real-time sensograms of eIPs conjugated and reference surfaces are given in Figure S11. The affinity calculations performed with the evaluation software revealed a *K*_d_ value of 6.48 × 10^–12^ M. Such a difference in the reported affinity between two detection platforms may be attributed to the different affinity calculation methods as well as working principles of transducers.

The in silico-designed eIPs accomplished 4 orders of magnitude lower limit of detection than a previous work in which a whole-adenovirus-imprinted polymeric material showed a detection limit of 8 × 10^6^ PFU mL^–1^ via an optical sensing mechanism.^40^ In the same work, the sandwich immunoassay developed for the adenovirus revealed an LOD of 3.23 × 10^6^ PFU mL^–1^ which is still 4 orders of magnitude higher than the LOD of the current work. In a recent work, Bajaj et al. reported SARS-CoV-2-imprinted nanopolymers utilized in a surface plasmon resonance (SPR)-based sensor for PoC diagnostics.^16^ The portable SPR sensor coupled with virus-imprinted MIPs achieved a detection sensitivity down to 10^5^ PFU mL^–1^. The virus-imprinted electrochemically synthesized polymeric materials were utilized for impedimetric detection of foot and mouth disease virus.^41^ The imprinted materials exhibited a measurement range of 4–75 ng mL^–1^ with a detection limit of 1.98 ng mL^–1^ and serotype specificity. Assuming a virus mass is 1 fg, this value is translated to 34.5 pM, which is much larger than the detection limit of the current work. A whole-virus-imprinted electrochemical sensor was utilized for the rapid detection of plant virus.^42^ A polypyrrole-based sensing platform achieved a detection limit of 0.41 pg mL^–1^ in diluted plant extract samples. Khan and colleagues fabricated a microfluidic sensing unit for PoC detection of H1N1 utilizing 2-amino-1,3,4-thiadiazole as the functional monomer for electrosynthesis of a virus-imprinted polymer film.^43^ The multichannel electrochemical sensor could detect H1N1 with high sensitivity (LOD: 9 TCID50/mL) and selectivity. In another work, a gas-responsive resonance light scattering biosensor was designed to be utilized in Hepatitis B virus detection, facilitating a reversible binding of the target virus.^44^ The regenerable sensing platform revealed an imprinting factor of 6.7 with great sensitivity (LOD: 1.9 pM). Imprinted biosensing agents were synthesized using metal organic frameworks and zinc acrylate, achieving fluorescence-based detection of Japanese encephalitis virus (JEV) as low as 13 pM in 20 min.^45^ In a recent work, a flexible polylactic acid-based electrospun membrane immunosensor was fabricated for optical detection of SARS-CoV-2 with a detection limit of 10 TU mL^–1^.^46^ The eIP-QCM revealed multiple magnitudes of an order lower LOD (10^2^ PFU mL^–1^, 0.2 fM) compared to the previous works since it combines the rational receptor design methods with the epitope-imprinting approach. Furthermore, the high sensitivity and compactness of eIP-QCM pave the way for its utilization as a PoC device in communities with limited resources.

Navakul and colleagues developed a Dengue virus-imprinted QCM sensor using graphene oxide as signal amplification agents to achieve an LOD of 0.58 PFU mL^–1^ and an investigation range of 10^0^–10^4^ PFU mL^–1^.^47^ Although the GO-enriched sensor provides a higher sensitivity, the detection range of the eIP-QCM sensor is broader with less cost and simplicity, as it does not require any nanomaterial enrichment.

### Selectivity and Cross-Reactivity of eIP-QCM

Selectivity of the eIP-QCM sensor toward HAdV was evaluated using control eIPs (Figure 4B). Control eIPs were synthesized for a 22-mer epitope derived from p53 protein via the solid-phase synthesis method. Following their synthesis, control eIPs were immobilized on a gold QCM substrate using a MUDA self-assembly monolayer and EDC–NHS coupling chemistry and exposed to HAdV solutions in buffer within the concentration range of 10^2^–10^7^ PFU mL^–1^. A significant difference between the binding behaviors of HAdV-specific eIPs and control eIPs was observed despite the resembling epitopes (i.e., both are peptides of 22 amino acids). The control eIPs lacked a concentration-dependent trend and showed distinctly lower signals throughout the detection range as opposed to HAdV-specific eIPs. The overall plot shows that although HAdV was bound to control eIPs to some extent, the interaction was unspecific and limited. Additionally, NIP particles synthesized without any template were tested for HAdV detection to confirm the selectivity of the eIPs. NIP-conjugated QCM platform showed a very limited frequency response toward HAdV insertion for the entire concentration range (10^2^–10^7^ PFU mL^–1^) with an imprinting factor of 14 (Figure S12). The observations highlight the selectivity of HAdV-specific computational eIP, leading to an efficient HAdV binding profile.

Real samples such as tap water and blood include many other binding species that may interfere with the detection of HAdV. In order to investigate the specificity of eIP-QCM, cross-reactivity against other pathogenic viruses such as HSV-1, SARS-CoV-2, and EAV was examined (Figure 4D). Each pathogen was injected onto eIP-QCM at a moderate concentration of 10^5^ PFU mL^–1^ in buffer. The lowest cross-reactivity was observed for EAV which is a viral pathogen for horses with a size of 40–60 nm. HSV-1 (ca. 130 nm) was another interferent agent showing a low binding response toward the eIP-QCM sensor. Such low cross-reactivities were expected for both HSV-1 and EAV due to their size and shape differences from HAdV. On the other hand, SARS-CoV-2 (70–90 nm) is similar to HAdV (90 nm) in terms of not only size but also shape as it carries knob-like structures called spikes. Because of the comparable size and shape of SARS-CoV-2 to HAdV, it showed a moderate level of cross-reactivity. However, eIP-QCM showed 3 times higher affinity toward HAdV than SARS-CoV-2 despite the resemblance. The investigations concluded that the cavities of the in silico-designed eIPs were highly specific for HAdV.

### Electrochemical Characterization of eIP Sensor

Two commonly used electrochemical analytical methods, namely, cyclic voltammetry (CV) and square-wave voltammetry (SWV), were employed for the investigation of the electrode surface for eIP immobilization and HAdV recognition. The largest current output for CV and SWV measurements was obtained for the bare electrode (Figure S13) as the ferri/ferrocyanide molecules could freely undergo redox reactions on the electrode surface. Following the eIP conjugation on the gold surface, the current signal was suppressed significantly (49.7%) since the surface was covered with polymeric particles hindering the access of the probe solution to the working electrode. The lowest current was recorded for HAdV binding to the cavities of eIPs as the sensor was covered with virus particles preventing the diffusion of redox moieties toward the gold surface. SW voltammogram depicted signal suppression from 1290.6 3 μA for bare to 648.3 and 344.6 μA for eIP-conjugated and HAdV-bound surfaces, respectively (Figure S13B). Further investigations were performed with fluorescence microscopy to depict the successful eIP immobilization on a gold wire electrode at 2× (Figure S13C) and 10× (Figure S13D) magnifications.

### Real Sample Analysis

The sensitive and selective detection of viral pathogens in water resources is crucial to provide clean water to people in vulnerable communities lacking a hygienic environment. Therefore, the computationally developed portable eIP-QCM sensor was tested for the detection of HAdV particles in tap water to investigate its applicability in real samples. In order to achieve this, HAdV-spiked tap water samples diluted with 10% PBS were injected onto the eIP-QCM sensor at a concentration range of 10^2^–10^7^ PFU mL^–1^. The frequency response was evaluated for 10 min, and Δ*F* values were calculated with respect to the reference signal obtained by the unspiked medium. A logarithmic regression analysis was performed for the experimental data of HAdV detection in tap water, revealing an *R*^2^ value of 0.98 (Figure 4E). A gradual increase of Δ*F* was observed with the increasing HAdV concentration in the tap water samples, and an LOD of 10^2^ PFU mL^–1^ was achieved within a wide detection range of 10^2^–10^7^ PFU mL^–1^.

Virus detection in health samples such as blood and serum is critical, especially in the case of a viral pandemic where a large number of specimens are required to be evaluated in a limited time and budget. The suitability of the miniaturized and economical eIP-QCM sensor was examined for such applications by evaluating HAdV detection in diluted human serum samples (Figure 4F). The logarithmic regression analysis revealed an *R*^2^ value of 0.96. In general, human serum samples provided higher sensor signals than PBS and tap water samples as they accommodate various molecules and proteins that unspecifically bind to the surface. A proportionally escalating sensor readout was realized for a wide working range of 10^2^–10^7^ PFU mL^–1^ with a detection limit of 10^2^ PFU mL^–1^. The portable eIP-QCM sensor achieved the same LOD of 10^2^ PFU mL^–1^ in complex media as it did in buffer medium. Such specific and selective virus recognition with a portable sensing device shows the potential applicability of eIP-QCM in real scenarios.

Obtaining reproducible sensor data in human serum in comparison to tap water is more challenging due to its complexity and higher amount of interferents. We approach these significant challenges by working with computationally designed eIPs and establishing new recipes that can provide much higher binding performance than the conventional counterparts. Moreover, the modification of synthetic receptors with certain molecules such as PEG can be taken further into account to prevent unspecific binding.

Other biosensors for pathogenic virus diagnostics are listed in Table S2 for comparison, clearly showing that the eIP-QCM sensor can achieve higher sensitivities than other recognition elements such as antibodies and aptamers thanks to its unique recipe optimized with molecular dynamics simulations.

Computational modeling provides a fast and cost-effective receptor design; however, it is still challenging to perform molecular dynamics simulations for an highly extended period of time and accounting for very complex media. Moreover, classical MD simulations do not account for bond formation or breakage and electron transitions which are needed for chemical reaction description. In spite of these challenges, computational simulations clearly improve the design of synthetic receptors, as we have demonstrated in our work. Further acceleration in MD-based receptor design can be achieved by the implementation of machine learning tools to overcome the present limitations.^48^

## Conclusions

In this work, a compact QCM sensor is conjugated with computationally designed epitope-imprinted polymers for the sensitive and rapid detection of HAdV in tap water and human serum.

The computationally constructed eIPs achieved around 2 times higher affinity than conventional eIPs with only one functional monomer, showing that the proposed computational strategy can lead to the design of smart MIPs utilizing less material while achieving a higher sensitivity. Furthermore, the eIP-QCM sensor could detect HAdV particles as low as 10^2^ PFU mL^–1^ (0.2 fM) in human serum and tap water samples within a wide working range of 10^2^ −10^7^ PFU mL^–1^ with remarkable specificity and selectivity. The combination of a portative QCM platform with in silico-designed epitope-mediated cost-effective synthetic receptors exhibited a promising future for the PoC detection of viral pathogens in water resources and body fluids.

## Supporting Information Available

Experimental details on reagents and chemicals, computational evaluations, adenovirus epitope synthesis, and characterization; preparation of virus samples, preparation of control viruses, characterization of eIP-QCM sensor, and electrochemical characterization; and results of computational simulations with candidate functional monomers, chemical structure and characterization of adenovirus epitope, FT-IR spectrum of computationally designed eIPs, optimization studies of sensor fabrication, and electrochemical characterization of the eIP sensor (PDF)
