# Supplementary material for: Computationally Designed Epitope-Mediated Imprinted Polymers versus Conventional Epitope Imprints for the Detection of Human Adenovirus in Water and Human Serum Samples
Source: ACS Sens. 2024 Mar 15;9(4):1831–41. doi: 10.1021/acssensors.3c02374 (PMC11059108; doi:10.1021/acssensors.3c02374)
Supplement: Supplementary file 1 — se3c02374_si_001.pdf [file se3c02374_si_001.pdf]

ACS SENSORS

SUPPORTING INFORMATION

# Computationally designed epitope-mediated imprinted polymers vs conventional epitope imprints for the detection of human adenovirus in water and human serum samples

Ekin Sehit[1,2], Guiyang Yao[1], Giovanni Battocchio[1], Rahil Radfar[1,2], Jakob Trimpert[3], Maria A. Mroginski[1], Roderich Süssmuth[1], Zeynep Altintas[1,2,4]*

[1] Institute of Chemistry, Technical University of Berlin, Straße des 17. Juni 124, Berlin 10623, Germany

[2] Institute of Materials Science, Faculty of Engineering, Kiel University, 24143 Kiel, Germany

[3] Institute of Virology, Free University of Berlin, 14163 Berlin, Germany

[4] Kiel Nano, Surface and Interface Science (KiNSIS), Kiel University, 24118 Kiel, Germany

*Corresponding author: Z. Altintas

E-mail: zeynep.altintas@tu-berlin.de | zeynep.altintas@tf.uni-kiel.de

TABLE OF CONTENTS

**Supplementary Experimental Section** ........ **S-3**
Reagents and chemicals ........ S-3
Computational evaluations ........ S-3
Adenovirus epitope synthesis and characterization........ S-3
Preparation of adenovirus samples........ S-3
Preparation of control viruses ........ S-4
Surface plasmon resonance-based affinity evaluation ........ S-4
Electrochemical characterization ........ S-4
**Supplementary Results** ........ **S-5**
Figure S1. Secondary structure analysis of HAdV fiber knob protein (PDB: 6HCN). ........ S-5
Figure S2. H-bonds formed between acrylic acid and the epitope during molecular dynamics simulation. ........ S-5
Figure S3. H-bonds formed between methacrylic acid and the epitope during molecular dynamics simulation. S-6
Figure S4. H-bonds formed between 4(5)-Vinylimidazole and the epitope during molecular dynamics simulation. ........ S-6
Figure S5. H-bonds formed between acrylamide and the epitope during molecular dynamics simulation. ........ S- 7
Figure S6. H-bonds formed between methacrylamide and the epitope during molecular dynamics simulation. S-7
Figure S7 Number of contacts for A) acrylic acid and B) methacrylic acid during molecular dynamics simulations. Salt bridges between acrylic acid and eptiope's C) lysine and D) serine residues. ........ S-8
Figure S8. A) Chemical structure of aspartic acid modified (shown in blue) adenovirus epitope. B) Gradient system for the peptide with their respective retention time and ESI-HR-MS data. UV-detection at λ = 210 nm; buffer A: 0.1% HCOOH in $H_2O$; buffer B: 0.1% HCOOH in MeCN. Size distribution of eIPs C) by intensity, and D) by number (n=3). E) Average zeta potential of eIPs. . ........ S-9
Figure S9. FT-IR spectrum of in-silico designed eIP. ........ S-10
Figure S10. HRTEM images of computationally designed HADV specific eIPs. ........ S-11
Table S1. Optimization of eIP concentration for sensor fabrication. ........ S-11
Equation S1. Sauerbrey equation for piezoelectric quartz crystals. ........ S-11
Figure S11. A) Real-time SPR sensogram for HAdV binding on eIPs modified and reference sensor surfaces. B) Concentration dependent response plot for affinity calculation. ........ S-12
Figure S12. Comparative analysis of eIPs and NIPs for HAdV detection on QCM platform. ........ S-12
Figure S13. The immobilization of eIPs on gold electrode and consecutive rebinding of adenovirus were confirmed with A) cyclic voltammetry and B) square-wave voltammetry techniques. The measurements were obtained using 10 mM $K_3(Fe(CN)_6)$ in 0.1 M KCL at room temperature. Fluorescent microscopy images of gold wire electrode after eIP conjugation at C) 2x and D)10 x magnifications. ........ S-13
Table S2. Virus biosensors listed for comparison. ........ S-13
**References** ........ **S-14**

## SUPPLEMENTARY EXPERIMENTAL SECTION

### Reagents and chemicals

3-aminopropyltriethyloxysilane (APTES) was purchased from Acros Organics (Germany). Acrylic acid, N-(3-Aminopropyl)methacrylamide hydrochloride (APMA), N-N'-Methylenebisacrylamide (BIS), N-isopropylacrylamide (NIPAM), N-tert-butylacrylamide (TBAAm), N′-tetramethylethylenediamine (TEMED), ammonium persulfate (APS), glutaraldehyde 25% aqueous solution (GA), human serum, bovine serum albumin (BSA), 11- mercaptoundecanoic acid (MUDA), N-hydroxysuccinimide (NHS), sodium acetate, ethanolamine, methanol, toluene, acetone, ethanol, and phosphate buffer saline (PBS) tablets were purchased from Sigma Aldrich (Taufkirchen, Germany). 1-Ethyl-3-(3-dimethylaminopropyl)carbodiimide hydrochloride (EDC-HCl) was received from Carl Roth (Karlsruhe, Germany). The QCM measurement unit openQCM-1 and QCM crystals were purchased from Novaetech S.r.l., Italy. Double-distilled ultrapure water produced by a Millipore Direct-Q® 3 UV (Millipore, Germany) was used throughout the study.

### Computational evaluations

The adenovirus epitope was taken from the crystal structure of HAdV fibre knob protein (PDB: 6HCN), located in the capsid with a residue sequence of AKLTLVLTKCGSQILATVSVLA (419-440) adopting a β -hairpin structure. The terminal ends are methylated. The protein was solvated in a water box and neutralized with two $Cl^-$ ions. All simulations were performed on GPUs with a 2 fs time step under periodic boundary conditions with the particle-mesh-Ewald method for electrostatic interactions, a cutoff of 12 Å for the van der Waals interaction and hydrogens constrained with the SHAKE algorithm. The setup was minimized for 40 ps, heated from 0 to 100 K (NVT) in 60 ps and then from 100 to 300 K (NPT) in 80 ps and finally pre-equilibrated at 300 K (NPT) for 120 ps. From the last pre-equilibrated structure, 1000 ns MD simulations were performed at 300 K (NVT). For functional monomer selection, the epitope structure resulting from equilibration was randomly surrounded by 10 copies of each of the functional monomer (acrylic acid, methacrylic acid, 4(5)-vinyl imidazole, acrylamide, methacrylamide) while solvated and neutralized. The H-bonds and salt bridges formed between the epitope and the monomer were monitored for performance evaluation. Two functional monomers demonstrating the highest number of bonds were further simulated with the epitope in combination while the epitope total monomer ratio remained as 1:20.

### Adenovirus epitope synthesis and characterization

To best of our knowledge, adenovirus epitope was synthesized in a research lab for the first time in this work using solid-phase peptide synthesis technique by following two major steps: (i) loading and (ii) coupling of Fmoc/tBu-protected amino acids [1].

(i)*Loading:* 1 g of 2-chloro-tritylchloride resin (CTC resin, 1.6 mmol $g^{-1}$) and 10 mL dichloromethane (DCM) were added to a 20 mL syringe reactor with frit and cap. The resin was preswollen for 30 min and the solvent was filtered. A mixture of the first amino acid Fmoc-Ala-OH (140 mg) and 5 equivalents of N,N-diisopropylamine (DIPEA) dissolved in 10 mL dry DCM was added to the resin. The syringe was shaken for 30 min at room temperature. The solution was filtered, and the resin was washed using N,N-dimethylformamide (DMF, 2 × 10 mL), DCM (2 × 10 mL). Capping was performed with DCM, methanol, and DIPEA 80:15:5 (2 × 10 mL, 10 min). After washing with DMF (5 × 10 mL), Fmoc-removal was achieved with 20% piperidine in DMF for 30 min.

*(ii)Coupling of Fmoc/$^t$Bu-protected amino acids:* To 1 gram of the resin (≈0.3 mmol $g^{-1}$ ), 4 equivalents of amino acid, 4 equivalents of TBTU (O-(benzotriazole-1-yl)-N,N,N′,N′-tetramethyluronium tetrafluoroborate) and 10 equivalents of DIPEA in DMF was added and shaken for 30 min. The adenovirus peptide with different amino acid sequences were synthesized using this protocol with an alternating sequence of Fmoc-deprotections and amino acid couplings. To orthogonally synthesize linear adenovirus peptide with aspartic acid modifications, the Boc-Ala-OH at N-terminal was used instead of Fmoc-Ala-OH and the Fmoc-Lys(Alloc)-OH was used instead of Fmoc-Lys(Ac)-OH. After removal of the Alloc group in the presence of $Pd(PPh_3)_4$ and phenylsilane, the Fmoc-Asp(tBu)-OH was coupled on the side chain.

After removal of the last Fmoc group, the resin was transferred to a 5 mL syringe with frit and cap. After addition of the cleavage cocktail (trifluoroacetic acid (TFA), $H_2O$, triethylsilane (TES), 95:2.5:2.5), the syringe was shaken for 1 h. The peptide was precipitated in ice cold diethyl ether and centrifuged. The supernatant was removed, and the precipitate was washed with diethyl ether twice. The peptide was resolved in MeCN/$H_2O$ (1:4) and lyophilized. Crude peptide variants were purified by reversed-phase high performance liquid chromatography (HPLC). Collected fractions containing the products were pooled and lyophilized to afford the adenovirus peptides as powder solid.

### Preparation of adenovirus samples

Human adenovirus serotype 5 was grown on HEK-293 (ATCC CRL-1573) cells and harvested 3 days after infection. Cells were lysed through 3 repeated freeze-thaw cycles, virus particles were purified by 2 rounds of cesium chloride (Merck, Darmstadt, Germany) density gradient centrifugation as previously described [2]. Cesium chloride was removed by gel filtration on Sephadex G-25 (Pharmacia, Uppsala, Sweden) equilibrated with injection buffer (3 mM KCl, 1 mM $MgCl_2$, phosphate buffered saline (PBS) and 10% glycerol). Subsequently, concentrated virus particle suspensions were passed through a 0.45 µm filter and stored in aliquots at -80 °C for further use. Virus titers were determined by titration of 10-fold serial dilutions on HEK-293 cells. Additionally, particles counts were assessed by spectrophotometry as described previously [3]. Concentrated particle suspensions contained 2–5 × $10^{11}$ particles per ml. Prior to further use, virus stocks were diluted to a final particle concentration of $1x10^9$ plaque forming unit (PFU) $mL^{-1}$ and fully inactivated by the addition of 0.5 % glutaraldehyde (Applichem, Darmstadt, Germany) in molecular grade water (Thermo Scientific, Waltham, MA, USA) and incubation for >48 hours at 8 °C.

## Preparation of control viruses

Equine arteritis virus (EAV) and Herpes simplex virus type 1 (HSV-1) were propagated on RK13 [ATCC CCL-37] and Vero [ATCC CCL-81] cells, respectively, using Dulbecco's Modified Eagle's Medium (DMEM) (PAN-Biotech, Germany) supplemented with 1% Penicillin/Streptomycin. Ultra-filtration of both viruses was carried out using Vivaspin ® 2 Centrifugal Concentrator (MWCO 50 KDa; Sartorius, Stonehouse, UK) according to manufacturer's instructions. [4] Briefly, cellular debris was removed by centrifugation for 5 min, 10000 g at 4 °C. Two steps of 2 ml virus stock centrifugation at 4°C (4000 g) for 20 minutes followed by 2 times washing steps with PBS were conducted using Vivaspin concentrator columns. A final volume of approximately 200 µL virus concentrate was collected and titrated on the respective cell line for each virus. Final concentration of $1x10^9$ PFU $mL^{-1}$ for HSV-1 and $1.3 \times 10^8$ PFU $mL^{-1}$ for EAV were achieved, prior to further use virus was inactivated by the addition of 25 % Glutaraldehyde to a final concentration of 0.5 % and incubation for >48 hours at 8 °C.

Severe acute respiratory coronavirus 2 (SARS-CoV-2) particles were obtained from passage 3 of an early 2020 SARS-CoV-2 B.1 outbreak isolate (BetaCoV/ Germany/ BavPat1/2020) propagated on Vero E6 cells. All work handling infectious SARS-CoV-2 was performed under appropriate BSL-3 safety conditions (Freie Universität Berlin, Institut für Virologie). VeroE6 cells (ATCC CRL-1586) were cultured in minimal essential medium (MEM; PAN Biotech, Aidenbach, Germany) supplemented with 10% fetal bovine serum (PAN Biotech, Aidenbach, Germany), 100 IU $mL^{-1}$ penicillin G and 100 µg $mL^{-1}$ streptomycin (Carl Roth, Karlsruhe, Germany). Virus particles were obtained 48 hours post infection of confluent Vero E6 cells. For virus harvest, cell culture flasks were frozen at -80°C and thawed to obtain crude lysate which was centrifuged for 10 min at 4°C to remove cellular debris and titrated to obtain virus titers on Vero E6 cells as previously described [2]. Virus containing supernatant was subsequently inactivated by 24 hours of incubation in 4% neutral buffered formaldehyde at room temperature and exported from the BSL-3 lab for further purification by ultracentrifugation using a sucrose gradient as previously described [5].

## Surface plasmon resonance-based affinity evaluation

Gold surface of sensor chip (Cytiva, Germany) was modified with 2mM 11-Mercaptoundecanoic acid solution and conjugated with HAdV-specific computational eIPs (2µL $min^{-1}$, 30 minutes) following the 4 minutes activation of surface with 0.2 M EDC and 0.05 M NHS mixture. The unoccupied sites on the sensor were blocked with 100 µg $mL^{-1}$ bovine serum albumin (BSA) and 0.1 mM ethanolamine treatment. HAdV samples were prepared in 10 mM phosphate buffer saline with 0.05% Tween for a concentration range of $10^3$ – $10^7$ pfu $mL^{-1}$ and injected to sensor for 9 minutes with 120 s of dissociation step. The resulting sensogram was evaluated with Biacore X100 evaluation software for steady-state affinity calculation.

## Electrochemical characterization

Cyclic voltammetry (CV) and square-wave voltammetry (SWV) techniques were employed for characterization of eIP conjugated sensor and consecutive HAdV binding event. The three-electrode setup including Pt counter electrode, Ag/AgCl reference electrode, and gold working electrode was utilized with PalmSens4 potentiostat (Belltec, Lüdenscheid, Germany). A potential range of −0.2 to 0.8 V with a scan rate of 0.05 V $s^{-1}$ was applied for CV measurements. SWV measurements were taken with a range of applied potentials of −0.3 to 0.8 V at an amplitude of 0.05 V and a frequency of 5 or 10 Hz. Gold electrodes were cleaned prior to each experiment following a previously reported method [6]. All measurements were taken in the presence of redox probe solution (10 mM $K_3Fe(CN_6)$ in 0.1 M KCl) at room temperature.

SUPPLEMENTARY RESULTS

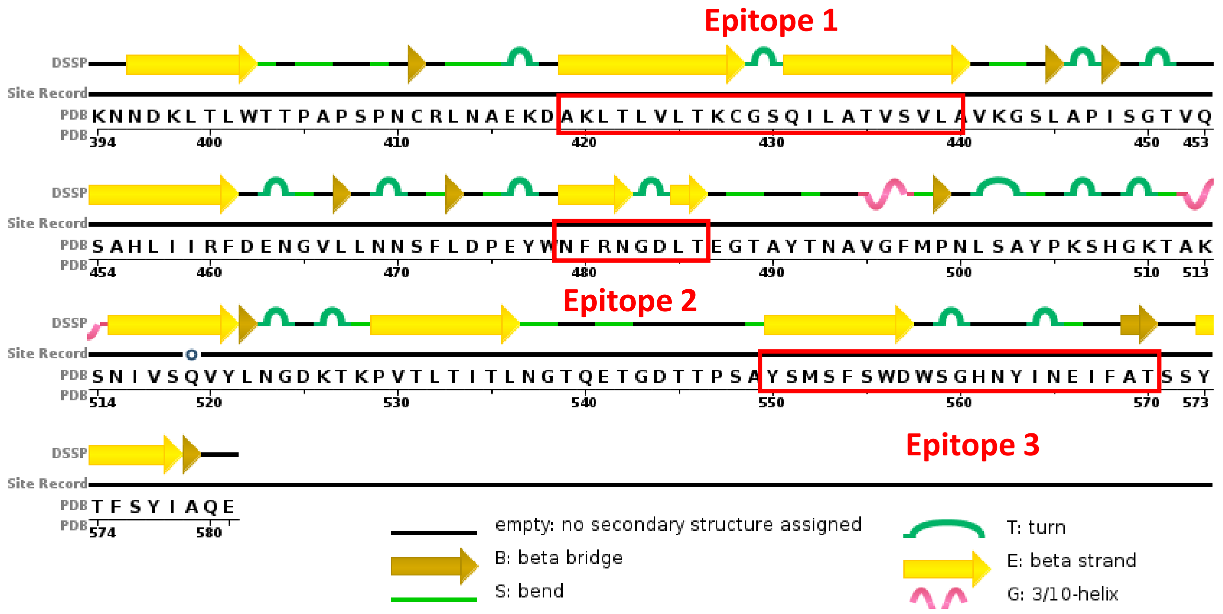


Figure S1. Secondary structure analysis of HAdV fiber knob protein (PDB: 6HCN).

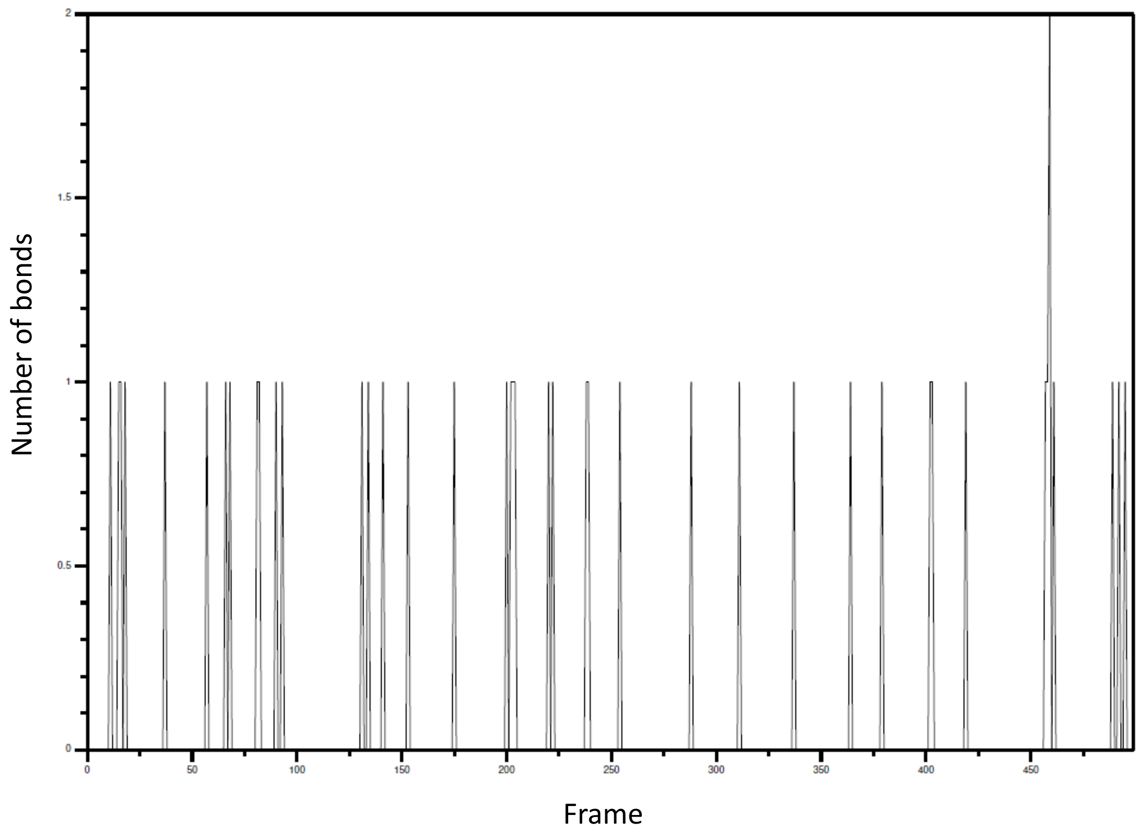


Figure S2. H-bonds formed between acrylic acid and the epitope during molecular dynamics simulation.

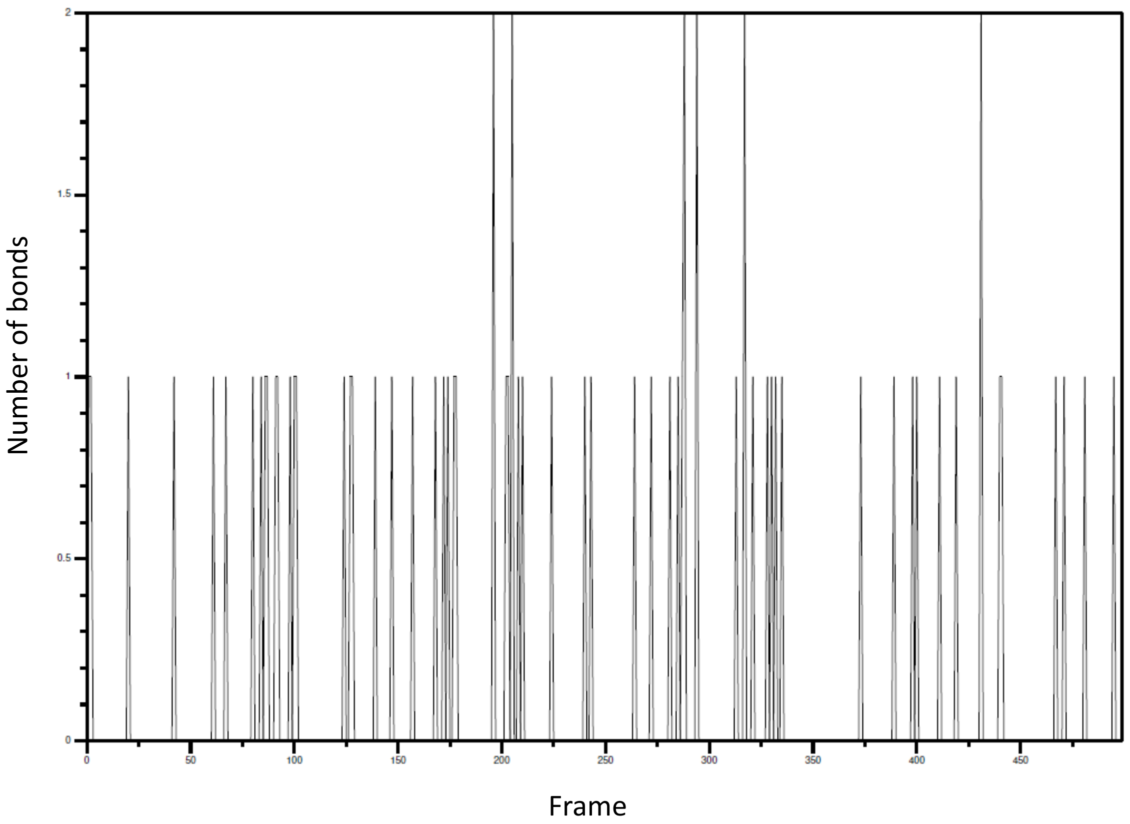


Figure S3. H-bonds formed between methacrylic acid and the epitope during molecular dynamics simulation.

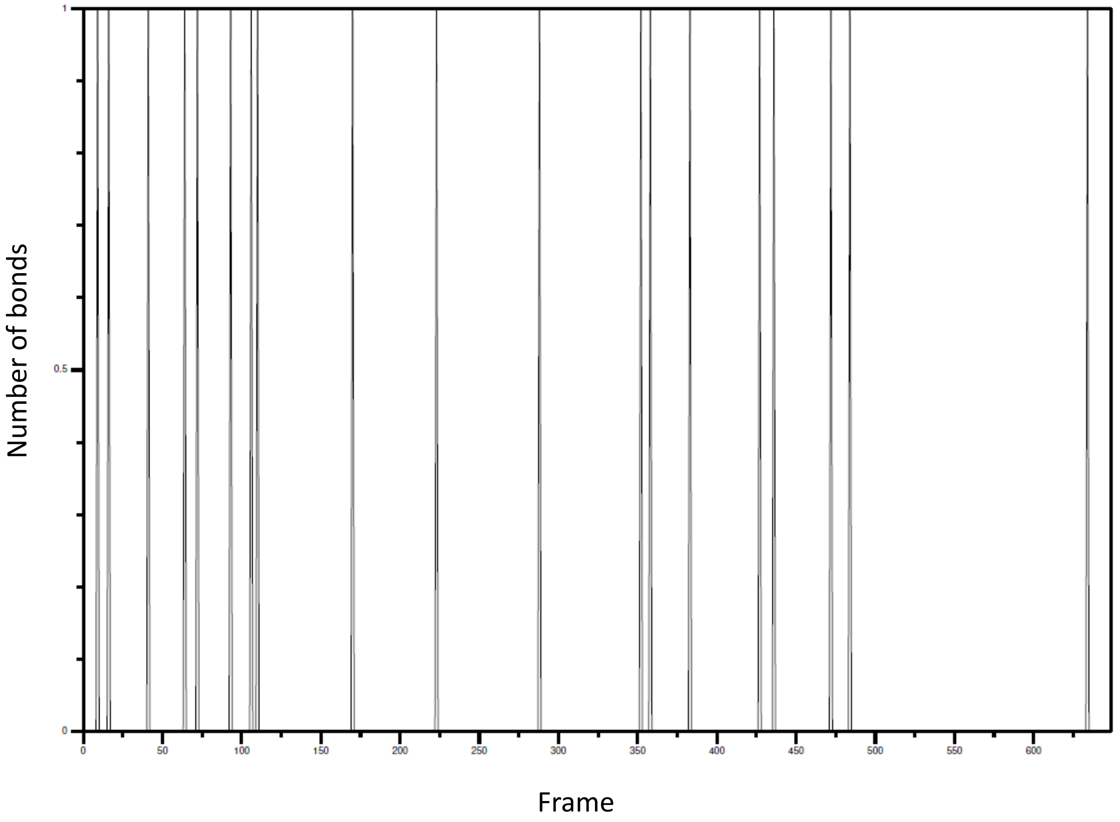


Figure S4. H-bonds formed between 4(5)-Vinylimidazole and the epitope during molecular dynamics simulation.

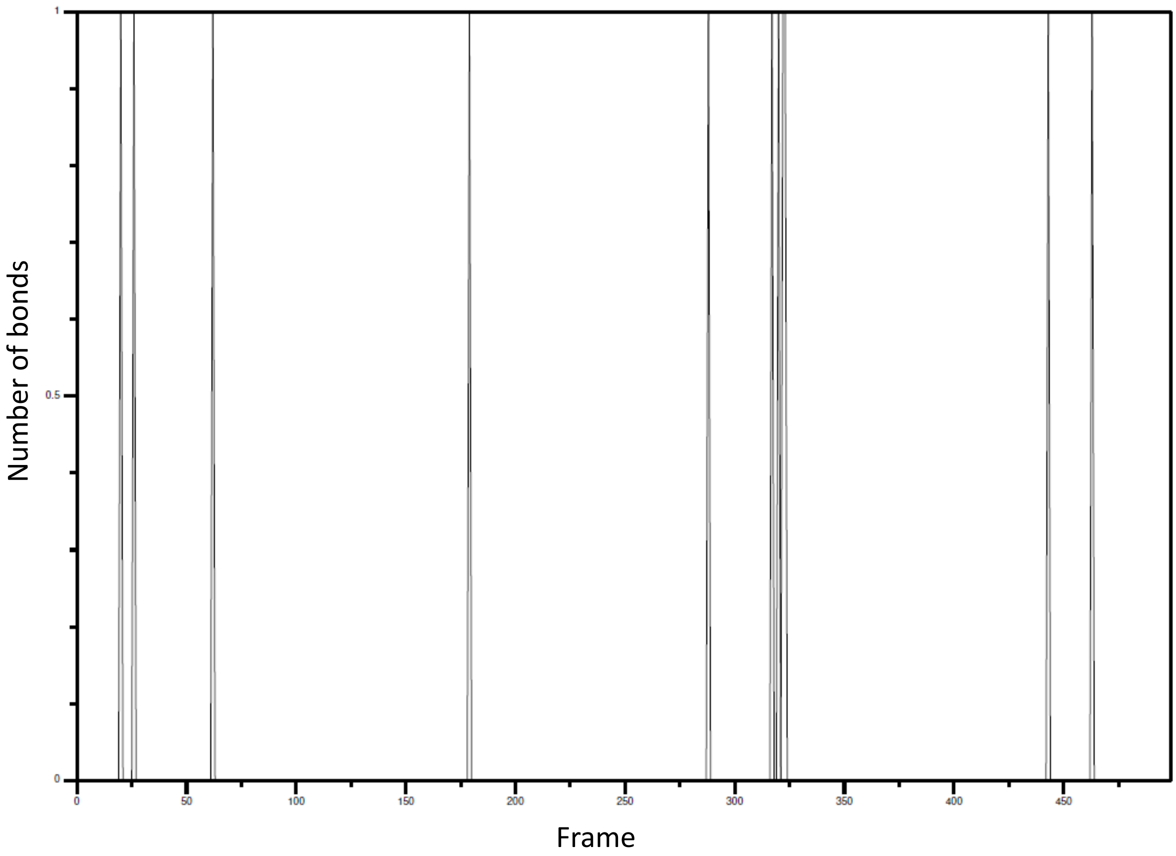


Figure S5. H-bonds formed between acrylamide and the epitope during molecular dynamics simulation.

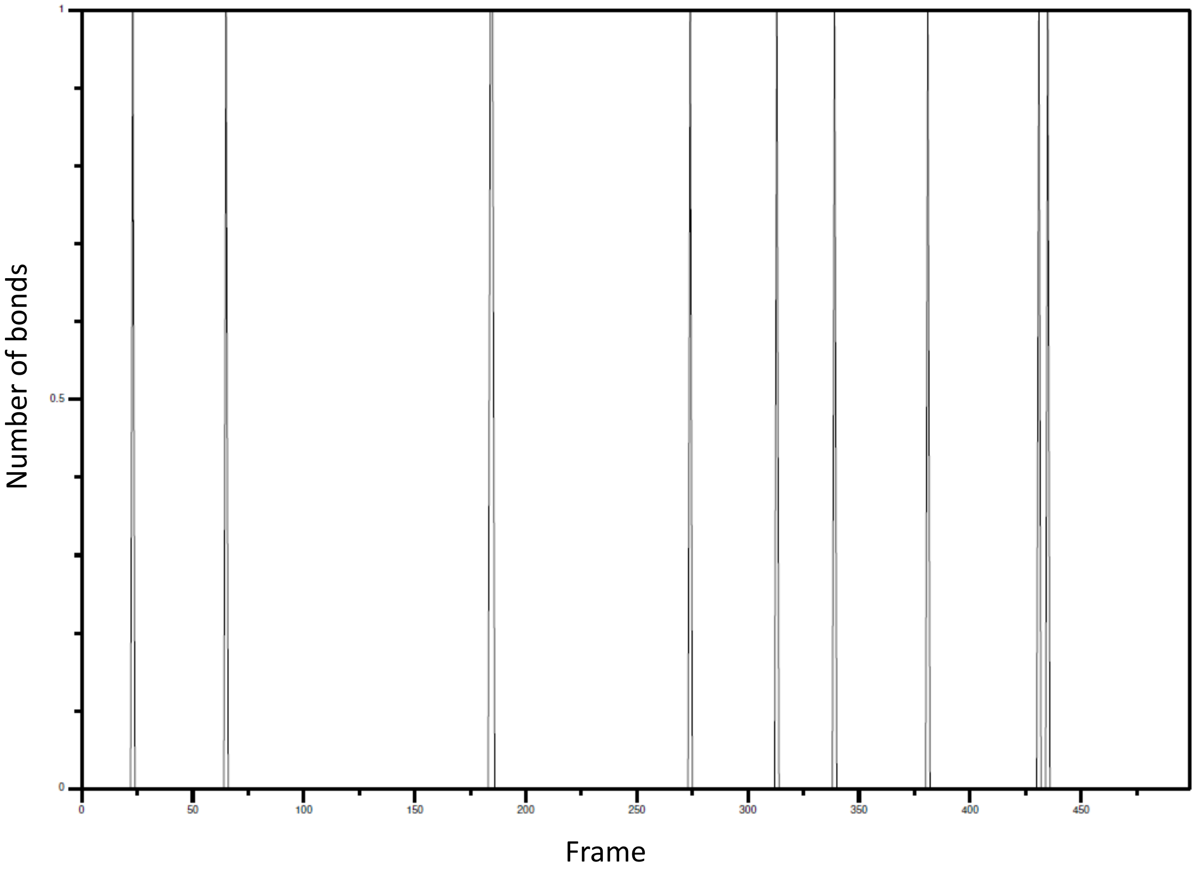


Figure S6. H-bonds formed between methacrylamide and the epitope during molecular dynamics simulation.

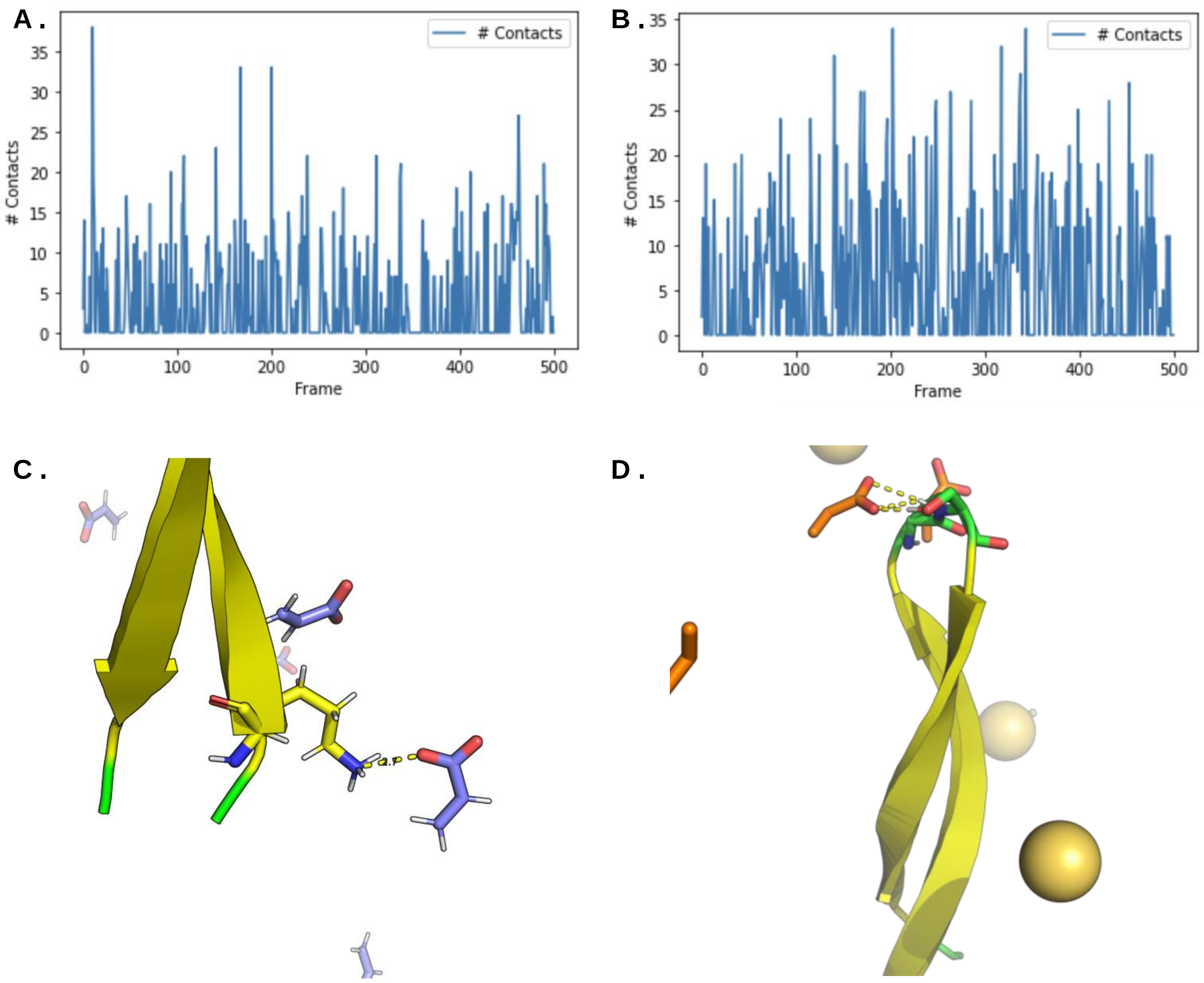


Figure S7. Number of contacts for A) acrylic acid and B) methacrylic acid during molecular dynamics simulations. Salt bridges between acrylic acid and epitope's C) lysine and D) serine residues.

A.

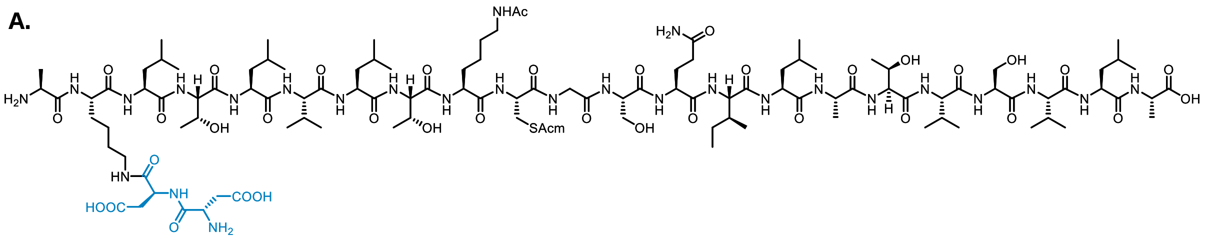

B.

| Peptide | Gradient | Retention time | ESI-HRMS calculated $[M+2H]^{2+}$ | ESI-HRMS found |
|---|---|---|---|---|
| adenovirus epitope | 20% to 100 % (0-10 min) | 6.21 min | $C_{113}H_{198}N_{28}O_{37}S^{2+}$ 1286.7169 | 1286.7208 |

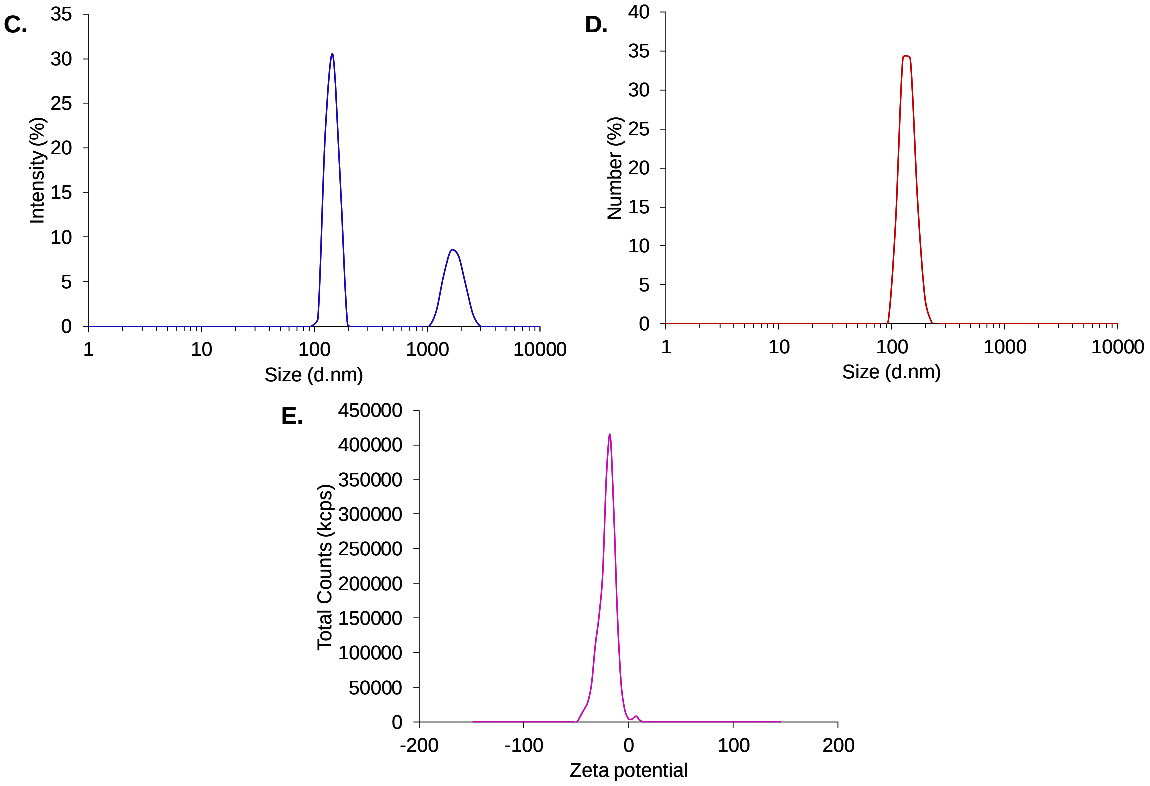


Figure S8. A) Chemical structure of aspartic acid modified (shown in blue) adenovirus epitope. B) Gradient system for the peptide with their respective retention time and ESI-HR-MS data. UV-detection at λ = 210 nm; buffer A: 0.1% HCOOH in $H_2O$; buffer B: 0.1% HCOOH in MeCN. Size distribution of eIPs C) by intensity, and D) by number (n=3). E) Average zeta potential of eIPs.

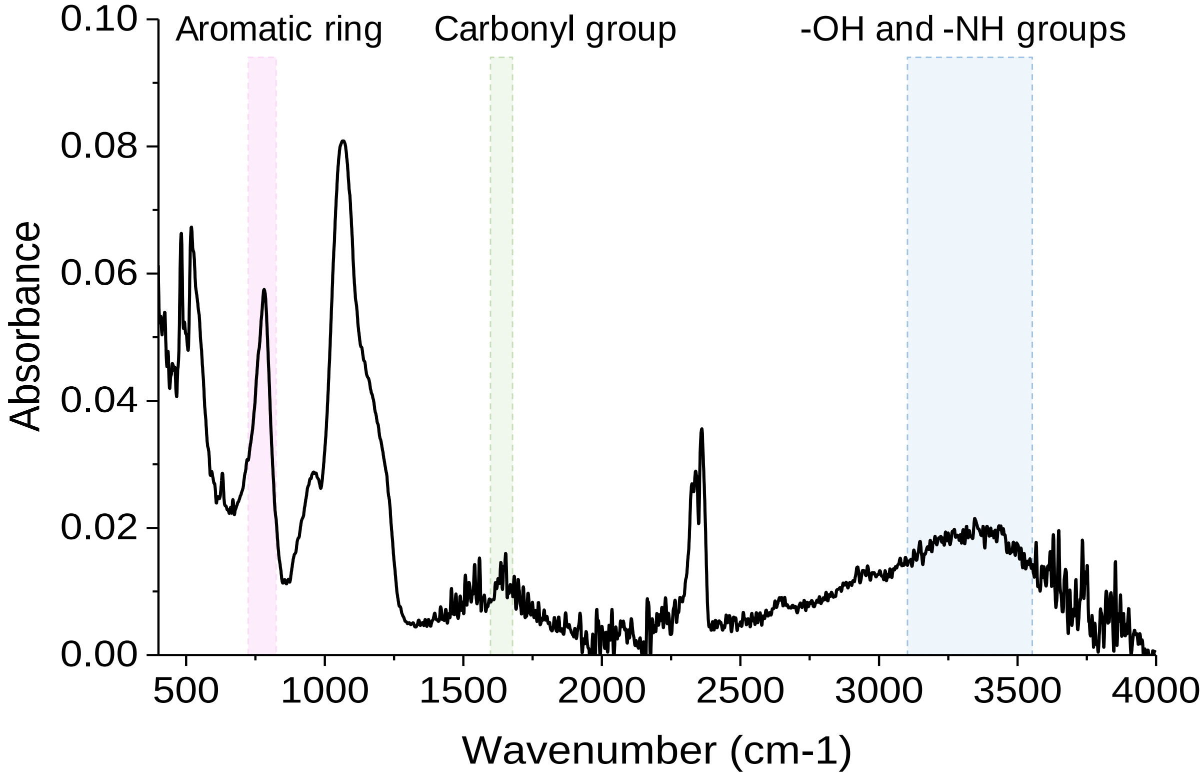


Figure S9. FTIR spectrum of in-silico designed eIP.

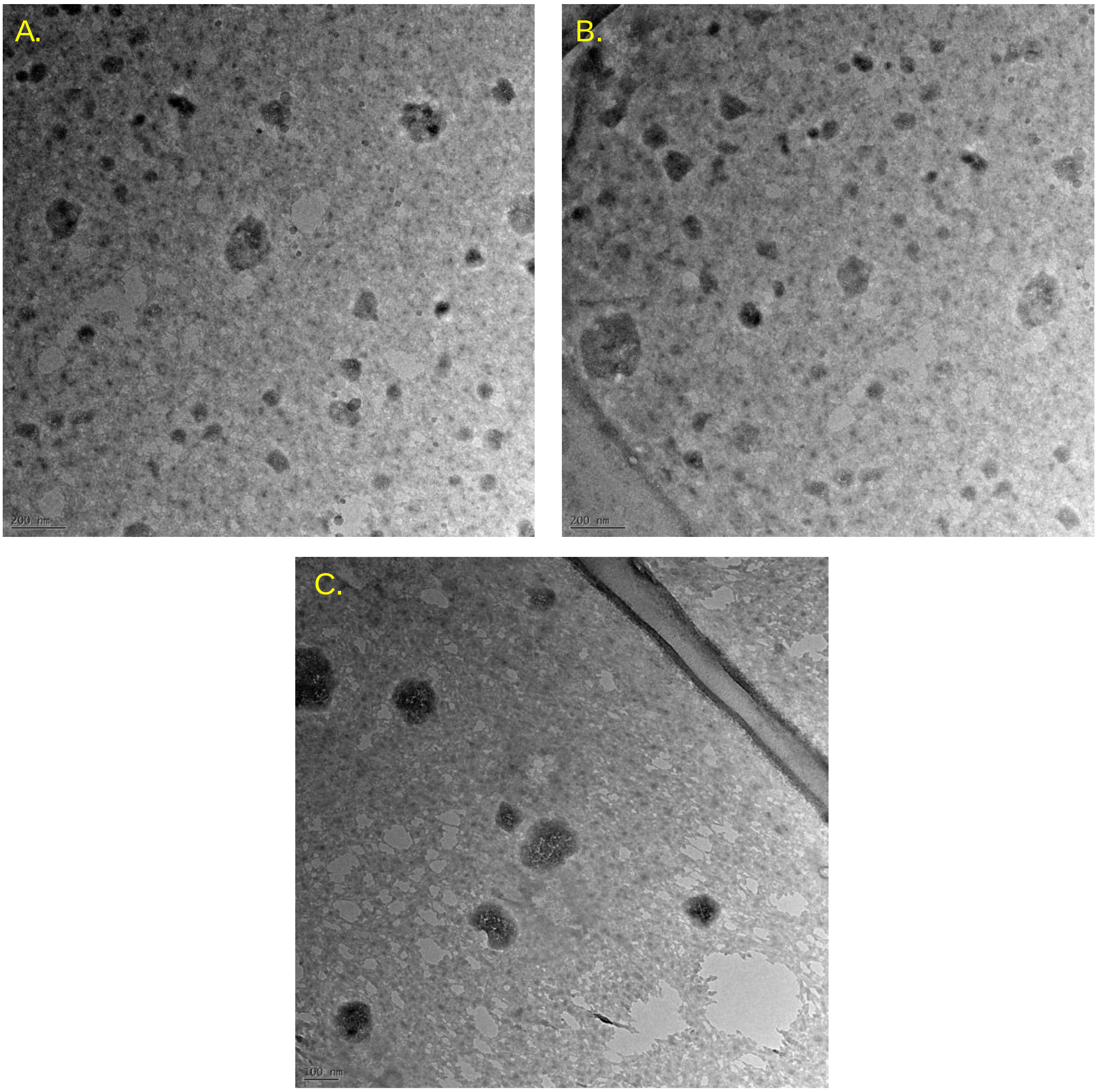


Figure S10. High resolution transmission electron microscopy images of computationally designed HADV specific eIPs.

**Table S1. Optimization of eIP concentration for sensor fabrication.**

| eIP concentration | Frequency decrease | Standard deviation |
|---|---|---|
| 0.5 mg mL$^{-1}$ | 3.66 Hz | ± 2.68 Hz |
| 1.0 mg mL$^{-1}$ | 60.11 Hz | ± 4.78 Hz |
| 1.5 mg mL$^{-1}$ | 18.78 Hz | ± 2.99 Hz |

$$\Delta m = -\frac{C}{n}\Delta F_n \quad \text{(S1)}$$

Equation S1. Sauerbrey equation for piezoelectric quartz crystals.

Δm is the mass of the added layer on the crystal surface per unit area, C is the mass-sensitivity constant, n is the number of the harmonic, and ΔF is the change in the resonance frequency at the n$^{th}$ harmonics. [7]

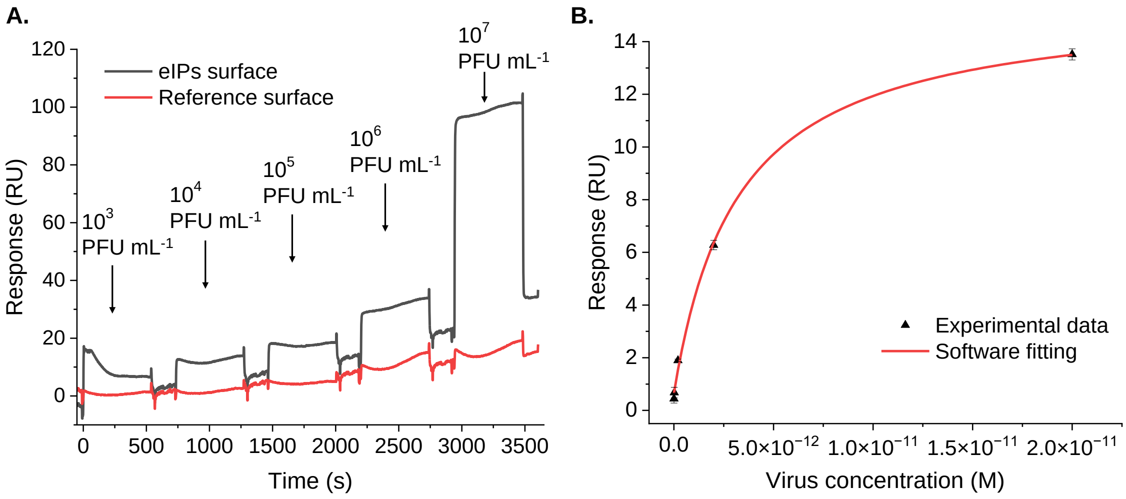


Figure S11. A) Real-time SPR sensogram for HAdV binding on eIPs modified and reference sensor surfaces. B) Concentration dependent response plot for affinity calculation.

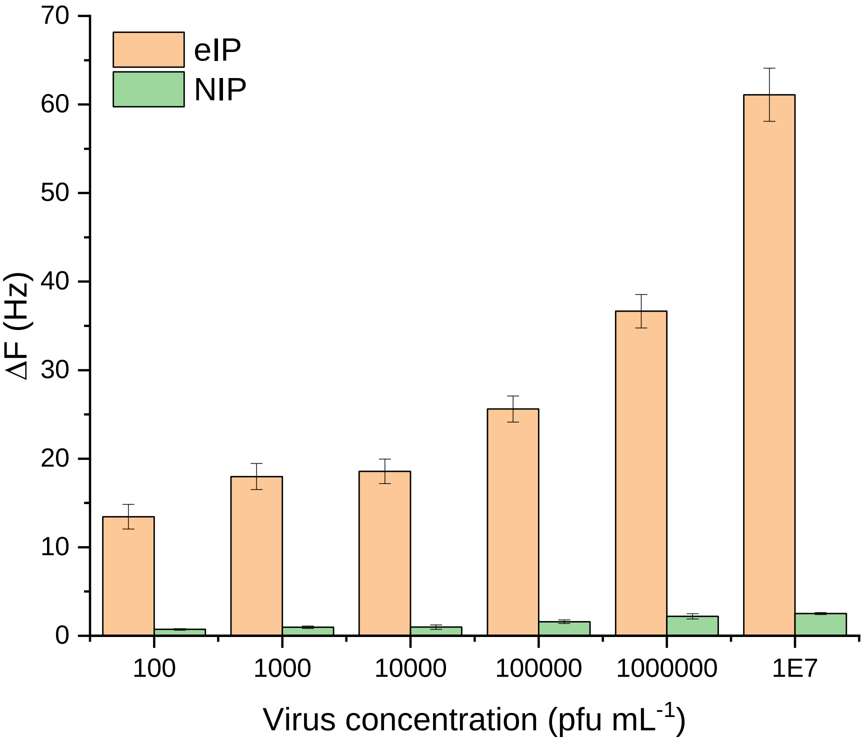


Figure S12. Comparative analysis of eIPs and NIPs for HAdV detection on QCM platform.

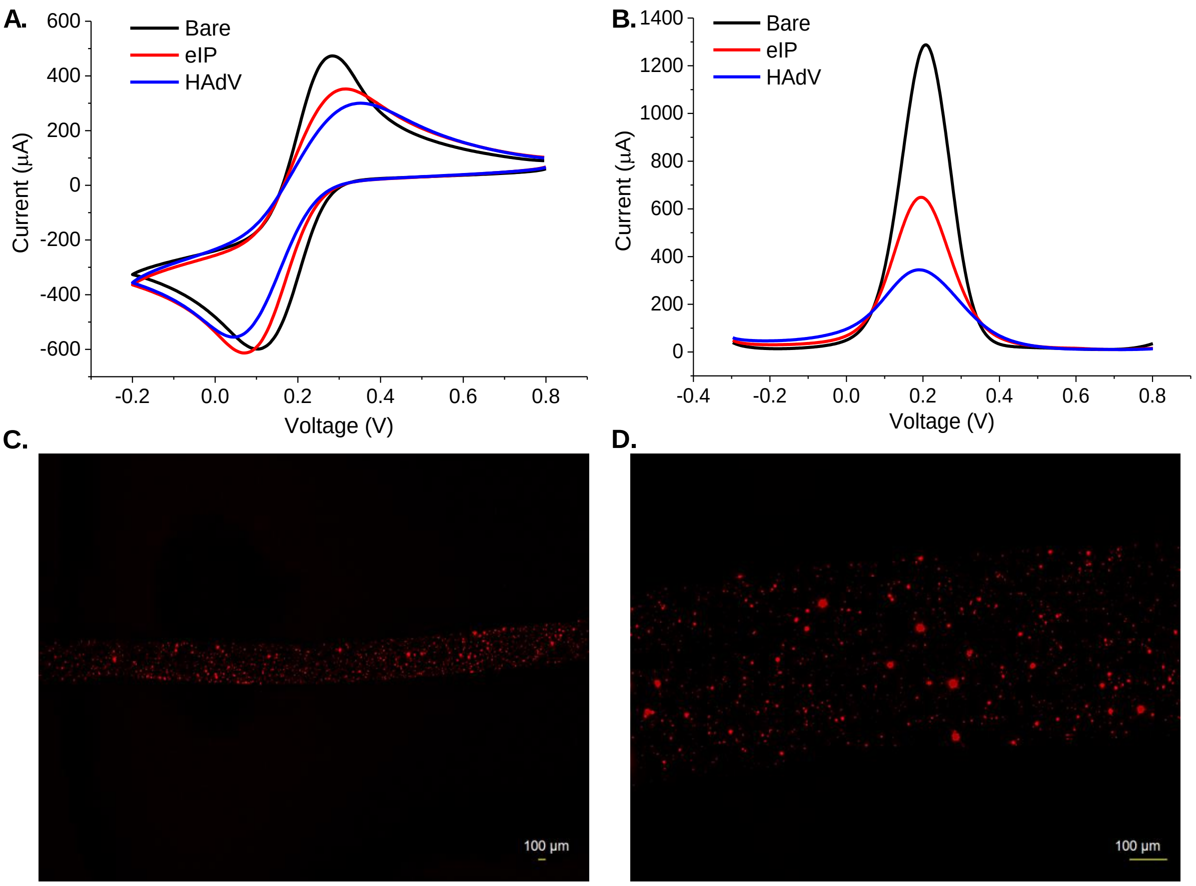


Figure S13. The immobilization of eIPs on gold electrode and consecutive rebinding of adenovirus were confirmed with A) cyclic voltammetry and B) square-wave voltammetry techniques. The measurements were obtained using 10 mM $K_3(Fe(CN)_6)$ in 0.1 M KCL at room temperature. Fluorescent microscopy images of gold wire electrode after eIP conjugation at C) 2x and D)10 x magnifications.

**Table S2. Virus biosensors listed for comparison.**

| Analyte | Receptor | Method | Detection range | LOD | Ref. |
|---|---|---|---|---|---|
| Measles virus | Antibody | Electrochemical | 10 – 100 μg mL$^{-1}$ | 6 μg mL$^{-1}$ | [8] |
| H1N1 gene | DNA | Optical | 10 pM – 100 nM | 0.152 pM | [9] |
| Rabies virus gene | RNA | Electrochemical | 0.145–25.39 ng μL$^{-1}$ | 0.104 ng μL$^{-1}$ | [10] |
| COVID-19 antibody | Antibody | Electrochemical | 4.68 – 300 ng mL$^{-1}$ | 4.68 ng mL$^{-1}$ | [11] |
| Hepatitis B virus gene | DNA | Optical | 0.05 – 10 pM | 30.15 fM | [12] |
| SARS-CoV-2 protease | Peptide ampiphiles | Optical | 15 – 30 nM | 15.7 nM | [13] |
| SARS-CoV-2 viral RNA | Peptide nucleic acid | Electrochemical | 1 fM – 1 nM | 0.38 fM | [14] |
| Adenovirus | eIP | Piezoelectric | 0.2 fM– 20 pM | 0.2 fM | This work |

REFERENCES


(1) Altintas, Z.; Takiden, A.; Utesch, T.; Mroginski, M. A.; Schmid, B.; Scheller, F. W.; Süssmuth, R. D. Integrated Approaches Toward High-Affinity Artificial Protein Binders Obtained via Computationally Simulated Epitopes for Protein Recognition. *Adv. Funct. Mater.* 2019, *29* (15), 1–11. https://doi.org/10.1002/adfm.201807332.

(2) Trimpert, J.; Adler, J. M.; Eschke, K.; Abdelgawad, A.; Firsching, T. C.; Ebert, N.; Thao, T. T. N.; Gruber, A. D.; Thiel, V.; Osterrieder, N.; et al. Live Attenuated Virus Vaccine Protects against SARS-CoV-2 Variants of Concern B.1.1.7 (Alpha) and B.1.351 (Beta). *Sci. Adv.* 2021, *7* (49), 1–10. https://doi.org/10.1126/sciadv.abk0172.

(3) Mittereder, N.; March, K. L.; Trapnell, B. C. Evaluation of the Concentration and Bioactivity of Adenovirus Vectors for Gene Therapy. *J. Virol.* 1996, *70* (11), 7498–7509. https://doi.org/10.1128/jvi.70.11.7498-7509.1996.

(4) Dumke, R.; Barron, M. de la C.; Oertel, R.; Helm, B.; Kallies, R.; Berendonk, T. U.; Dalpke, A. Evaluation of Two Methods to Concentrate SARS-CoV-2 from Untreatedwastewater. *Pathogens* 2021, *10* (2), 195. https://doi.org/10.3390/pathogens10020195.

(5) Cavanagh, D. *SARS- and Other Coronaviruses*; Cavanagh, D., Ed.; Humana Press, 2008; Vol. 454. https://doi.org/10.1142/9789814366922_0005.

(6) Sehit, E.; Drzazgowska, J.; Buchenau, D.; Yesildag, C.; Lensen, M.; Altintas, Z. Ultrasensitive Nonenzymatic Electrochemical Glucose Sensor Based on Gold Nanoparticles and Molecularly Imprinted Polymers. *Biosens. Bioelectron.* 2020, *165*, 112432. https://doi.org/10.1016/j.bios.2020.112432.

(7) Plikusiene, I.; Maciulis, V.; Ramanavicius, A.; Ramanaviciene, A. Spectroscopic Ellipsometry and Quartz Crystal Microbalance with Dissipation for the Assessment of Polymer Layers and for the Application in Biosensing. *Polymers (Basel).* 2022, *14*, 1056. https://doi.org/10.3390/polym14051056.

(8) Mayall, R. M.; Smith, C. A.; Hyla, A. S.; Lee, D. S.; Crudden, C. M.; Birss, V. I. Ultrasensitive and Label-Free Detection of the Measles Virus Using an N-Heterocyclic Carbene-Based Electrochemical Biosensor. *ACS Sensors* 2020, *5* (9), 2747–2752. https://doi.org/10.1021/acssensors.0c01250.

(9) Zhou, H.; Bu, S.; Xu, Y.; Xue, L.; Li, Z.; Hao, Z.; Wan, J.; Tang, F. CRISPR/Cas13a Combined with Hybridization Chain Reaction for Visual Detection of Influenza A (H1N1) Virus. *Anal. Bioanal. Chem.* 2022, *414* (29–30), 8437–8445. https://doi.org/10.1007/s00216-022-04380-1.

(10) Challhua, R.; Akashi, L.; Zuñiga, J.; Beatriz de Carvalho Ruthner Batista, H.; Moratelli, R.; Champi, A. Portable Reduced Graphene Oxide Biosensor for Detection of Rabies Virus in Bats Using Nasopharyngeal Swab Samples. *Biosens. Bioelectron.* 2023, *232*, 115291. https://doi.org/https://doi.org/10.1016/j.bios.2023.115291.

(11) Manshadi, M. K. D.; Mansoorifar, A.; Chiao, J. C.; Beskok, A. Impedance-Based Neutralizing Antibody Detection Biosensor with Application in SARS-CoV-2 Infection. *Anal. Chem.* 2023, *95* (2), 836–845. https://doi.org/10.1021/acs.analchem.2c03193.

(12) Cheng, R.; Tong Li, L.; Huang, M.; Zhu, F.; Li, Q.; Liu, H.; Gao, J.; Hui Zhao, X.; Kang Luo, F.; Wang, J. Highly Sensitive Plasmonic Biosensor for Hepatitis B Virus DNA Based on the Surface Etching of the Active

Helical Gold Nanorods. *Chem. Eng. J.* 2023, *468*, 143627. https://doi.org/https://doi.org/10.1016/j.cej.2023.143627.

(13) Jin, Z.; Li, Y.; Li, K.; Zhou, J.; Yeung, J.; Ling, C.; Yim, W.; He, T.; Cheng, Y.; Xu, M.; et al. Peptide Amphiphile Mediated Co-assembly for Nanoplasmonic Sensing. *Angew. Chemie* 2023, *135* (4), 1–9. https://doi.org/10.1002/ange.202214394.

(14) Li, Y.; Zhao, S.; Xu, Z.; Qiao, X.; Li, M.; Li, Y.; Luo, X. Peptide Nucleic Acid and Antifouling Peptide Based Biosensor for the Non-Fouling Detection of COVID-19 Nucleic Acid in Saliva. *Biosens. Bioelectron.* 2023, *225*, 115101. https://doi.org/https://doi.org/10.1016/j.bios.2023.115101.
